# Evolution of Patient and Public Involvement and Engagement in Health‐Related Research: A Concept Analysis

**DOI:** 10.1111/jan.70140

**Published:** 2025-08-16

**Authors:** Wenze Lu, Yan Li, Catherine Evans, David Currow, Jonathan Bayuo, Tingyu Zheng, Zhihui Lu, Mengqi Li, Julie Wray, Janelle Yorke

**Affiliations:** ^1^ School of Nursing The Hong Kong Polytechnic University Hong Kong China; ^2^ Faculty of Nursing, Midwifery and Palliative Care King's College London London UK; ^3^ Flinders Ageing Alliance Flinders University Bedford Park South Australia Australia; ^4^ School of Nursing, Midwifery, Social Work and Social Sciences University of Salford Salford UK; ^5^ Division of Nursing, Midwifery and Social Work Faculty of Biology, Medicine and Health Sciences, University of Manchester Manchester UK

**Keywords:** conceptual models of nursing, patient advocacy, patient participation, patient perspectives, PPIE

## Abstract

**Aims:**

To clarify the definition and evolution of Patient and Public Involvement and Engagement (PPIE) and identify its attributes, antecedents, and consequences in health‐related research.

**Design:**

This study follows Rodgers' evolutionary concept analysis with a seven‐step framework.

**Methods:**

Datasets were searched using terms related to PPIE and key categories (i.e., attributes, antecedents, and consequences). Data were sourced from CINAHL, PsycInfo, Scopus, PubMed, and Web of Science covering publications from inception to October 31, 2024. Document titles, abstracts, and keywords were manually screened to identify relevant studies for full‐text review.

**Results:**

A total of 1751 documents were screened, resulting in 38 eligible studies included in the final analysis. PPIE has evolved from a narrow focus on patient inclusion and participation, where patients had minimal influence on research and researchers resisted sharing control of research, to a collaborative model emphasising sustained partnerships, shared contributions, equitable power distribution, and active involvement across research stages. This shift has been driven by research innovation, a growing emphasis on healthcare equity and patient‐centred care, technological advances, and stakeholder advocacy (e.g., patients, funders, ethics committees). While PPIE enhances research relevance and impact, barriers, such as resource constraints, power imbalances, patient limited research capabilities and increased researcher workload persist. Facilitators, such as training programmes, standardised guidelines, flexible arrangements and transparent communication can enable meaningful partnerships.

**Conclusion:**

The concept of PPIE is evolving toward greater clarity and consistency in research, positioning patients and the public as active, essential contributors rather than passive participants. Barriers and facilitators were identified to inform its utilisation in research.

**Impact:**

This study clarifies the conceptual ambiguities of PPIE, informs theory development, and provides actionable insights. Healthcare and nursing researchers can draw on its findings to utilise PPIE to enhance collaborative and inclusive research practices that align with the needs of patients and the public.

**Reporting Method:**

This study adheres to the PRISMA (2020) reporting guidelines for systematic reviews.

**Patient or Public Contribution:**

One of our co‐authors is a patient with lived experience of cancer, who contributed valuable comments and suggestions to enhance this paper.

## 1 Introduction

### 1.1 Overview of the Problem Area

Health researchers often undertake research independently throughout the entire process from identifying research questions and designing methodologies to implementing interventions and sharing research findings, with limited active collaboration with patients and the public (Biddle et al. 2021; Ocloo et al. 2021). While health researchers have extensive research expertise and theoretical knowledge, they cannot fully represent the views and perspectives of patients and the public (Grover et al. 2022). Consequently, research may fail to align with the needs of patients and communities, undermining its practical relevance and contributing to inefficiencies in service delivery and resource allocation (Barello et al. 2012; Kwon et al. 2018). In response, Patient and Public Involvement and Engagement (PPIE) has emerged as a common approach to innovative research practice in health, integrating the views, insights, and experiences of patients and the public into research processes to enhance the relevance, applicability, and impacts of studies (National Institute for Health and Care Research (NIHR) 2021). While increasingly mandated by funding bodies and research institutions (NIHR 2021), the conceptual foundations of PPIE remain inconsistently defined and frequently conflated with other related terms. This conceptual ambiguity presents challenges for PPIE implementation and may undermine the theoretical coherence and operational consistency of PPIE in research.

### 1.2 Conceptual Background

Many research institutions, including the NIHR, advocate the value of PPI, which is defined as research conducted ‘with’ or ‘by’ members of the public, rather than ‘to,’ ‘about,’ or ‘for’ them (NIHR 2021). This concept emphasises active collaboration between researchers and patients or the public at various stages of the research process, rather than merely recruiting them as participants. The NIHR (2022) has updated PPI to PPIE, introducing the element of engagement, which involves two‐way interactions such as sharing research findings with the public, facilitating public access to the benefits, and raising awareness through events. Other studies have similarly conceptualised PPIE as involving a broader range of stakeholders, including patients, carers and community members acting as co‐creators of research (Bensenor et al. 2022; Keane et al. 2023; Lu et al. 2025).

PPIE is often confused or conflated with related concepts, such as patient‐public engagement, shared decision‐making, participatory health research, co‐design, co‐creation and co‐production (DeLacy 2021; Li et al. 2024; Locock and Boaz 2019; Slattery et al. 2020; Vinnicombe et al. 2023). For example, participatory health research involves working with those affected by an issue to foster education and drive action (Cargo and Mercer 2008). Co‐design often involves research users in the early planning stages of a study (Slattery et al. 2020). Co‐production generally refers to a process that engages patients and professionals as equal partners in the development of healthcare services, products, and interventions (DeLacy 2021). These terms to some extent share fundamental principles with PPIE but differ in scope, focus, and application, particularly regarding who should be involved, at what stages of the research process, and the extent of collaboration in health‐related research. Another ambiguity stems from confusion between tokenistic involvement and genuine collaboration. Some studies misidentify minimal consultation as PPIE, while others promote deeper engagement but lack operational clarity (Bergholtz et al. 2024). Additionally, the boundary between PPIE contributors and research participants is often blurred. While research participants are typically enrolled in studies to provide data and are subject to ethical protocols, PPIE contributors may be involved in activities such as co‐developing research protocols and designs or informing dissemination strategies without being enrolled as participants or contributing data directly (Ocloo and Matthews 2016; Spencer et al. 2023; Staley et al. 2021).

### 1.3 The Importance of the Study

Inconsistencies in definitions, overlapping terminologies, and a lack of conceptual clarity of PPIE may lead to confusion among researchers and practitioners, resulting in misalignment with established PPIE practices or incomplete implementation in research contexts (Bergholtz et al. 2024; Forbat et al. 2009; Staley et al. 2021). Given the increasing emphasis on the use of PPIE in health research, a rigorous conceptual clarification of PPIE is essential to facilitate its coherent understanding and effective application.

Concept analysis, originally proposed by Wille (1982) and further developed through Rodgers' evolutionary model (Rodgers 1989), offers a systematic method for addressing the conceptual ambiguity of PPIE in health research. This approach facilitates the clarification of PPIE concepts, including the identification of key features of PPIE, PPIE antecedents, and PPIE consequences, while distinguishing PPIE from related terms and accounting for contextual and temporal variation (Duffy et al. 2025). Clarifying these components of PPIE can contribute to a clear and consistent understanding of the PPIE concept, support PPIE theoretical development in health research, guide the effective application of PPIE in practice, and inform PPIE‐related education and training (He et al. 2024).

## 2 Research Aim and Questions

This study aimed to analyse the concept of PPIE in health‐related research by clarifying its definition and evolution, attributes (i.e., key features), antecedents (i.e., the underlying factors for its emergence), and consequences (i.e., benefits, barriers and facilitators in its utilisation). The research questions were formulated as follows:

Research Question 1 (RQ1): How is the term ‘PPIE’ defined and how has it evolved over time in health‐related research?

Research Question 2 (RQ2): What are the key attributes and antecedents of PPIE in health‐related research?

Research Question 3 (RQ3): What are the main consequences of PPIE in health‐related research?

## 3 Methods

### 3.1 Design

Rodgers' evolutionary concept analysis (Rodgers 1989), a robust methodological framework designed to analyse concepts that are dynamic and fluid, was employed in this study. It acknowledges that concepts are not fixed; instead, they evolve over time (Tofthagen and Fagerstrøm 2010). The philosophical foundation of Rodgers' evolutionary concept analysis method is relativism, which contends that knowledge and truth are shaped by social and cultural contexts, such as language, history, and power relations (Beckwith et al. 2008).

Rodgers' evolutionary concept analysis method includes a seven‐step analytical framework:

1. identifying the core concept of interest (i.e., PPIE in this study),
2. identifying surrogate terms and relevant uses of the concept,
3. selecting an appropriate realm for data collection,
4. identifying the attributes of the concept,
5. identifying the contexts, antecedents and consequences of the concept,
6. identifying related concepts, and.
7. writing a model case of the concept (Rodgers 1989).

The report of findings adhered to the Preferred Reporting Items for Systematic Reviews and Meta‐Analyses (PRISMA) 2020 guidelines to maintain transparency and methodological rigour throughout the study.

### 3.2 Surrogate Terms of the Concept

Previous research has shown that the terms, “patient and public involvement”, “co‐design”, “co‐creation”, and “co‐production” have often been used interchangeably in health‐related research to express overlapping ideas of PPIE (DeLacy 2021; Locock and Boaz 2019; Slattery et al. 2020; Vinnicombe et al. 2023). Accordingly, we identified these four terms as surrogate terms for PPIE in this study for subsequent data collection. Other terms (e.g., patient participation) that refer to limited or passive forms of involvement, such as providing informed consent, completing questionnaires, or participating in isolated consultations that do not meet the similarity criteria for PPIE, and fail to reflect the dual emphasis on both patients and the public were excluded from the surrogate terms.

### 3.3 Data Sources and Collection

A systematic search was conducted using Boolean operators'AN' and'O' across multiple databases, including the Cumulative Index of Nursing and Allied Health Literature (CINAHL), PsycINFO, SCOPUS, PubMed, and the Web of Science. To ensure a comprehensive search encompassing all elements of Rodgers' evolutionary concept analysis (e.g., concept, attributes, antecedents, consequences), we incorporated relevant synonyms and alternative terms and focused on titles, abstracts, and keywords of the literature published on or before 31 October 2024. The specific search terms used are presented in Table 1. In alignment with the third step of Rodgers' evolutionary concept analysis method (Rodgers 1989), we established clear inclusion and exclusion criteria for data selection, as outlined in Table 2.

**TABLE 1:** Key search terms.

Key search term 1	“patient and public involvement and engagement” OR “public and patient involvement and engagement” OR “patient involvement and engagement” OR “patient engagement and involvement” OR “public involvement and engagement” OR “public engagement and involvement” OR “Co‐design” OR “Co‐production” OR “Co‐creation” OR “Patient and public involvement” OR “public and patient involvement”
AND	
Key search term 2	“Concept” OR “Notion” OR “Construct” or “Principle” or “Framework” or “Model”
AND	
Key search term 3	“Attribute” or “Characteristic” or “Trait” or “Feature” or “Aspect” or “Dimension” or “Element” or “Component”
AND	
Key search term 4	“Antecedent” or “influence” or “factor” or “variable” or “Catalyst” or “Cause” or “Precondition”
AND	
Key search term 5	“Consequence” or “Outcome” or “Result” or “Effect” or “Impact” or “Implication” or “benefit” or “facilitator” or “barrier” or “challenge” or “obstacle”

**TABLE 2:** Criteria for inclusion and exclusion.

Criteria	Inclusion	Exclusion
Literature type	Peer‐reviewed journal articles and grey literature	N/A
Language	English	Non‐English
Date range	Published on or before 31 October 2024	N/A
Geographical area	No geographical limitations	N/A
Context	Health‐related research	Non‐health settings
Concept	PPIE and its surrogate terms and those shown in Key Search Term 1	N/A
Contents	Documents containing all the following elements: concepts, attributes, antecedents, and consequences	Documents mentioning only one or some of these elements, but not all

### 3.4 Data Extraction and Screening

**FIGURE 1:**
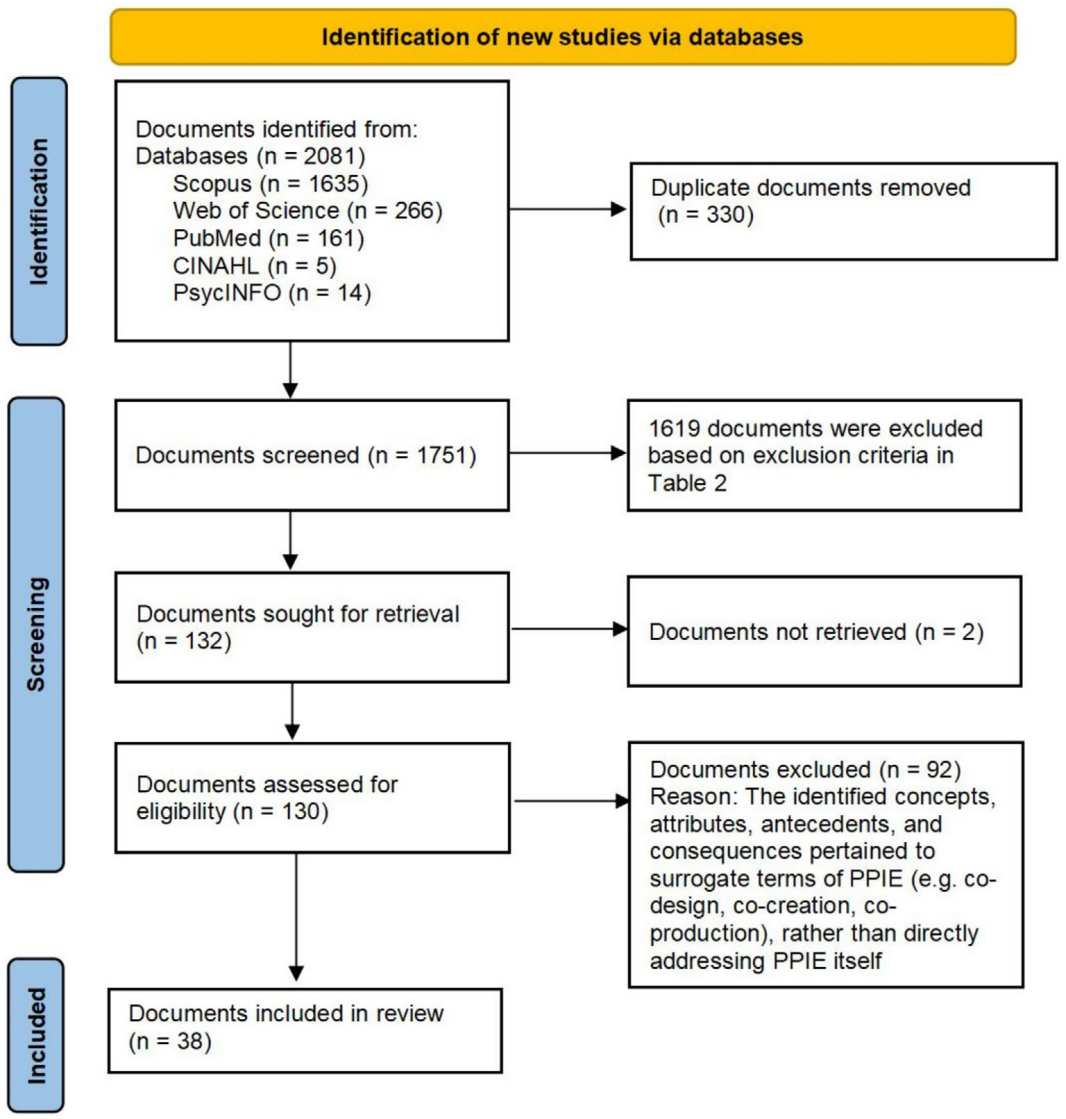
PRISMA flow diagram.

To facilitate retrieval, duplication checks, and screening decisions, all documents retrieved from the databases including peer‐reviewed articles, reviews, book chapters, and other relevant publications were exported to EndNote. The use of the term ‘documents’ aligns with the terminology used by the databases (e.g., Scopus and Web of Science). These documents were independently screened by two co‐authors in accordance with predefined inclusion and exclusion criteria. Documents that met the criteria were reviewed in full, and discrepancies were resolved through discussion with a third researcher. Relevant data were then manually extracted into summary tables for analysis. The tables captured information on PPIE definitions, attributes, antecedents, and consequences. Additionally, contextual elements such as country, setting, study design, and any illustrations of PPIE development or evolution were documented. Weekly meetings were conducted by the research team to ensure the reliability and accuracy of the data screening and extraction processes. Any disagreements were resolved through consensus among the research team. A PRISMA flow diagram is shown in Figure 1, indicating the number of documents identified, included, and excluded, along with the reasons for exclusion.

## 4 Overview of the Concept

**FIGURE 2:**
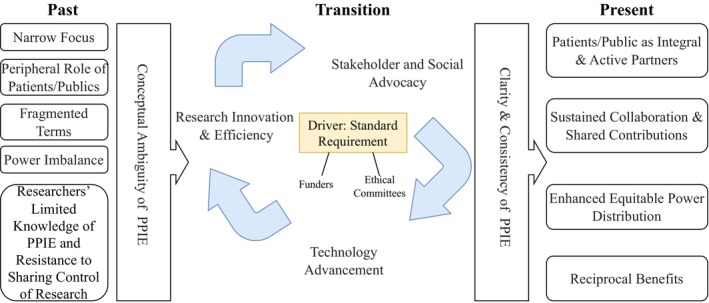
Evolution of the PPIE concept from past to present. (This figure illustrates the transition of the PPIE concept from its past state to its present form, highlighting its current key features advocated by researchers, patients, and the public, as well as the drivers facilitating its shift in health‐related research).

The study aims to address three research questions: (1) how the term PPIE has been defined and evolved over time; (2) what the core attributes and antecedents of PPIE are; and (3) what consequences of PPIE have been reported in health‐related research. A total of 38 documents were included, categorised as follows: 18 research articles (5 mix‐methods studies, 5 quantitative studies, 8 qualitative studies), 1 methodological study, 6 review articles, 12 commentaries, and 1 book chapter. These documents were published between 2016 and 2024, with the majority originating from the United Kingdom (UK) (*n* = 27), other European countries (*n* = 6), Canada (*n* = 1), the United States (U.S.) (*n* = 1), Australia (*n* = 1), Brazil (*n* = 1), and Pakistan (*n* = 1). The documents were reviewed, analysed, and synthesised to answer these questions. Generally, PPIE utilisation was identified across diverse healthcare contexts, including digital health technologies for neurological conditions such as dementia, community‐based ageing research addressing frailty in older adults, the management of non‐communicable diseases such as diabetes, the development of targeted digital applications for specific populations (e.g., youth mental health support), and its integration into drug development and regulatory processes. Across the documents analysed for this study, individuals with lived experience, particularly patients, were the most frequently involved stakeholders in PPIE, followed by carers, community members, healthcare professionals (e.g., clinicians, nurses, coordinators), researchers, advocates, charity representatives, and drug developers. The overall findings, including the definition, antecedents, attributes, and consequences of the PPIE concept, are presented in Appendix S1. Figure 2 presents an overview that highlights the evolution of the concept from past to present, grounded in the documents analysed.

### 4.1 Definition of PPIE and Its Evolution

The most frequently referenced concept of PPIE (*n* = 20) originates from the UK, which defines ‘Involvement’ as research being carried out ‘with’ or ‘by’ members of the public, rather than ‘to’, ‘about’, or ‘for’ them (NIHR 2021); and defines ‘Engagement’ as the provision and dissemination of information and knowledge about research to patients and the public (NIHR 2021). The term ‘public’ encompasses a broad spectrum of stakeholders who will benefit from the research, including patients, potential patients, and individuals directly affected by the research topic, such as caregivers, family members, and community members with relevant lived experience (Gray et al. 2021; Karlsson and Janssens 2023). Throughout the research process, these individuals can also be collectively referred to as public contributors (Keane et al. 2023). PPIE moves beyond simple consultation and passive involvement; it emphasises deeper partnerships that prioritise collaboration and actively empower individuals to influence and shape decision‐making processes (Wyatt et al. 2024). It also relates to co‐constructed, two‐way knowledge exchange and mutual benefit realisation (Aiyegbusi, Cruz Rivera, et al. 2023; Di Lorito et al. 2024).

PPIE can take place at specific stages of the research process (Hough et al. 2024; Fedorowicz et al. 2022) or extend across the entire research cycle (Hanrahan et al. 2024; Clark et al. 2021). The concept of PPIE did not vary significantly across different study contexts. However, in the UK, the concept is notably comprehensive and consistent. Other countries adopt more specific or narrower interpretations of PPIE. For example, in Brazil, the focus is on ensuring that participants act as co‐applicants across all research stages, emphasising shared decision‐making in project development (Bensenor et al. 2022). In Denmark, PPIE is primarily understood as the co‐development of healthcare services and research with users of those services (Karlsson and Janssens 2023). Canada and Pakistan conceptualise PPIE as a mechanism to advance health and healthcare research, with a strong focus on treating patients as partners in the research process rather than as passive subjects or mere data sources (Rolfe et al. 2018; Tolppa et al. 2024).

Studies suggest that PPIE has evolved from a *narrow focus* on patient inclusion and participation to a broader approach emphasising partnership and collaboration throughout key research stages (Clark et al. 2021; Bensenor et al. 2022; Zeissler et al. 2024). In the past, researchers *had limited knowledge of PPIE* and often resisted *sharing power* and *control of research* with patients and the public (Boaz et al. 2016; Chew‐Graham 2016; Hawkes et al. 2023). The increasing emphasis from stakeholders, especially international funding bodies and ethical committees requiring PPIE as a condition for research or grants, as well as the advancement of digital health technologies and research innovation, drives a more comprehensive and standardised PPIE concept in health‐related research (Aiyegbusi, Cruz Rivera, et al. 2023; Hough et al. 2024; Weiler‐Wichtl et al. 2023). PPIE now promotes patients and the public as *active and integral partners*, fostering *sustained collaboration and more balanced power dynamics* that *benefit* both researchers and public contributors (Hawkes et al. 2023; Karlsson et al. 2024).

Our study identified three prevalent terms related to PPIE: co‐design, co‐production, and co‐creation. These terms collectively encapsulate the core principles of PPIE, emphasising inclusivity, shared ownership, and the meaningful engagement of diverse stakeholders. The earlier *inconsistent and ambiguous use of terms* such as co‐design, co‐production, and co‐creation tends to transition toward more unified and standardised terminology, including ‘patient and public involvement’ and, more recently, PPIE, which explicitly incorporates both involvement and engagement (Hanrahan et al. 2024; Di Lorito et al. 2024; Karlsson and Janssens 2023). This transition has brought *greater clarity* to the practical implementation of PPIE, addressing concerns such as who should be involved, the scope of involvement and engagement, and the specific stages of the research process where activities should occur. The increasing precision has facilitated the development of structured and transparent approaches to PPIE, enabling researchers and stakeholders to utilise it more consistently and effectively. See Figure 2 for the evolution of the PPIE concept.

### 4.2 Attributes and Antecedents of PPIE

#### 4.2.1 Key Attributes of PPIE

Attributes are defined as the fundamental features that characterise a given concept. Five primary attributes of PPIE were identified:

1. Empowered public contributors with shared contributions, such as co‐applicants, co‐authors, co‐designers, and active partners, rather than passive subjects or participants (Bensenor et al. 2022; Spencer et al. 2023).
2. Sustained collaboration and partnership between public contributors and researchers (Hawkes et al. 2023; Karlsson et al. 2024).
3. Equitable power distribution (Aiyegbusi, McMullan, et al. 2023; Fedorowicz et al. 2022).
4. Reciprocal benefits grounded in mutual respect and shared exchange (Hough et al. 2024; Jameson et al. 2023; Zeissler et al. 2024).
5. Proactive involvement and engagement (Aiyegbusi, Cruz Rivera, et al. 2023; Hilton et al. 2024).

#### 4.2.2 Key Antecedents of PPIE

The antecedents of PPIE refer to the foundational conditions and driving factors that are necessary for its emergence (Rolfe et al. 2018). The study identified five primary antecedents.

1. Inequalities in access to healthcare. Persistent inequalities in healthcare access and outcomes highlight the need for PPIE to address disparities by involving marginalised populations in shaping more inclusive and equitable healthcare and research systems (El‐Nayir et al. 2024; Hough et al. 2024).
2. Increasing recognition of patient‐centered care. The growing focus on patient‐centered care and the need for research tailored to local contexts highlight a research paradigm in aligning healthcare and research practices with the real‐world priorities and experiences of patients (Karlsson and Janssens 2023; Fedorowicz et al. 2022).
3. Advocacy from stakeholders. The increasing emphasis from international funding bodies, policymakers, and research organisations on involving patients and the public lays a foundation for the emergence of PPIE to promote accountability, transparency, and societal relevance in healthcare and research (Aiyegbusi, Cruz Rivera, et al. 2023; Hough et al. 2024; Norrie et al. 2022). The global movement toward empowering patients to play active roles in healthcare and research supports PPIE as a mechanism to foster collaboration and co‐create practical, patient‐driven results (El‐Nayir et al. 2024; Hough et al. 2024).
4. Advances in digital health technology. The advances of digital health technologies foster innovations that are user‐centered, convenient, accessible, and responsive to the diverse needs of populations, creating opportunities to apply PPIE more effectively (Aiyegbusi, Cruz Rivera, et al. 2023). For example, mobile health applications and online patient portals facilitate real‐time communication, reducing barriers such as mobility limitations and geographic isolation while enabling patients to engage on their own terms (Lu et al. 2024; Weiler‐Wichtl et al. 2023). Digital technologies also support personalised engagement by leveraging patient‐generated data to tailor interventions (Micklewright et al. 2024) and allow individuals from underrepresented or marginalised communities to engage in research without the logistical and financial burdens often associated with in‐person participation (Aiyegbusi, Cruz Rivera, et al. 2023).
5. Research outcomes with limited effectiveness and impact. The limitations of traditional research processes, such as restrictive eligibility criteria, resource waste, and irrelevant findings, highlight the need for PPIE to address these inefficiencies by making research more inclusive, relevant, and impactful (Bensenor et al. 2022).

### 4.3 Consequences of PPIE

In this study, consequences refer to the benefits of PPIE on research, as well as the barriers and facilitators influencing its utilisation in research.

#### 4.3.1 Benefits of PPIE

The benefits of PPIE were manifested across three dimensions.

1. Research enhancement. More than 20 studies collectively demonstrate that PPIE enhances research relevance, quality, accessibility, ethical standards, and credibility by incorporating novel and diverse perspectives and better addresses the priority needs of patients and the public. PPIE also amplifies research impact through enhanced dissemination of findings and more effective clinical application (Hawkes et al. 2023; Karlsson and Janssens 2023). PPIE optimises resource utilisation and cost efficiency while minimising procedural variations and facilitating the early identification of potential challenges (Aiyegbusi, McMullan, et al. 2023). Increased public contributor satisfaction creates a positive feedback loop, which could improve recruitment rates for subsequent research initiatives (Hough et al. 2024).
2. Researcher development. For researchers, PPIE serves as a catalyst for innovation and professional growth. It facilitates a paradigm shift toward more inclusive research practices, fostering open‐minded attitudes toward and conversations with the public (Hough et al. 2024). Such a transformation in research culture promotes collaborative partnerships and enhances investigator expertise (Bensenor et al. 2022; Karlsson et al. 2024).
3. Patient and public empowerment. PPIE activities enhance the self‐efficacy of public contributors, develop their research competencies, and promote their active engagement in societal matters (El‐Nayir et al. 2024; Wyatt et al. 2024). The integration of diverse perspectives helps mitigate social inequalities and supports the advancement of inclusive healthcare practices (Hawkes et al. 2023).

#### 4.3.2 Barriers to PPIE Utilisation

Staff shortages and high turnover within research teams and PPIE groups, such as occupational mobility in personnel responsible for facilitating involvement, and the replacement of public contributors, have been identified as barriers to maintaining continuity and stability in PPIE processes within health‐related research (Hawkes et al. 2023). In some low‐income countries, limited emphasis on PPIE and lower levels of public health literacy prevent PPIE from functioning effectively (Aiyegbusi, Cruz Rivera, et al. 2023). Additionally, the legacy effects of researcher‐led or health professional‐led approaches may perpetuate unequal power dynamics and entrenched group identities, reducing PPIE to a superficial form of involvement, such as a tokenistic or box‐ticking exercise (Hough et al. 2024; Spencer et al. 2023).

Barriers specific to researchers include the perception of PPIE as an additional workload (Rolfe et al. 2018). The critical evaluation of PPIE practices remains under‐developed (El‐Nayir et al. 2024), with a lack of standardised guidelines for effective implementation (Karlsson and Janssens 2023). Limited knowledge, experience, and reference cases constrain researchers in navigating complex management challenges, such as maintaining long‐term partnerships, accommodating diverse individual needs, and managing participant attrition (Hawkes et al. 2023). Without effective collaboration with mediating institutions, researchers may also struggle to engage underserved populations (e.g., individuals from low‐income communities, ethnic minorities, or rural areas), compromising research representativeness and increasing the risk of bias (Karlsson et al. 2024).

Patients and members of the public, on the other hand, face their own set of challenges, such as stigma‐related concerns, lack of confidence and research capabilities, and limited interest in sharing personal experiences (Hough et al. 2024). Language and cultural differences, combined with the inherent complexity, nuances, and extended duration of research projects, often lead to misunderstandings and misjudgments among patients (Gray et al. 2021; Karlsson and Janssens 2023; Norrie et al. 2022). Research is driven by many factors, including a set of conventions which need to be understood in order to optimally engage the public and patients.

#### 4.3.3 Facilitators of PPIE Utilisation

Establishing clear protocols and role definitions for PPIE will facilitate the research preparation phase (Moult et al. 2023). Strategic partnerships with various institutions, particularly community organisations, can enhance participant recruitment while promoting inclusivity and diversity (Hawkes et al. 2023). Establishing trust‐based relationships with public contributors and fostering safe, supportive environments are essential prerequisites for meaningful PPIE (Karlsson and Janssens 2023). Training programmes for both researchers and participants, along with the formation of PPIE groups led by skilled, experienced, and open‐minded coordinators, are critical to PPIE utilisation (Hawkes et al. 2023). Standardised guidelines and frameworks provide a foundation for the consistent implementation of PPIE (El‐Nayir et al. 2024; Hough et al. 2024).

Facilitators in the implementation phase focus on three key components: flexible, context‐specific arrangements; transparent communication; and equitable power dynamics (Hawkes et al. 2023; Hough et al. 2024; Karlsson et al. 2024). Flexibility involves hybrid participation models that integrate digital health technologies for both online and in‐person engagement. Context‐specific approaches address diverse population needs; for instance, people with dementia are likely to benefit from accessible approaches that are aligned with their individual needs, while ethnic minority groups may engage more effectively in informal, culturally sensitive settings. Transparent communication requires comprehensive documentation, regular feedback mechanisms, and periodic adjustments. Bi‐directional feedback loops ensure public contributors stay informed while providing continuous input. Equitable power dynamics, fostered through respect and recognition, are also important for sustaining meaningful involvement throughout the study (Rolfe et al. 2018; Hough et al. 2024). Establishing mutual trust is also mentioned as a facilitator, as it helps maintain ongoing relationships (Jameson et al. 2023) and enhances understanding and engagement among underrepresented groups (Forbat et al. 2024).

In the evaluation phase, facilitators of PPIE utilisation include not only formal evaluation protocols and standardised reporting tools, but also practices that acknowledge and strengthen the role of public contributors. These include providing financial reimbursement for contributors' time and expenses (Moult et al. 2023) and using accessible formats to share research findings, such as infographics, summary videos, or illustrated reports, that make the outcomes of PPIE visible and understandable to participants and wider audiences (Hawkes et al. 2023; Polanco et al. 2022). Sustained engagement following the completion of research has also been identified as important for reinforcing trust and maintaining collaborative relationships, which support the continued integration of PPIE into future research activities (Karlsson et al. 2024).

**FIGURE 3:**
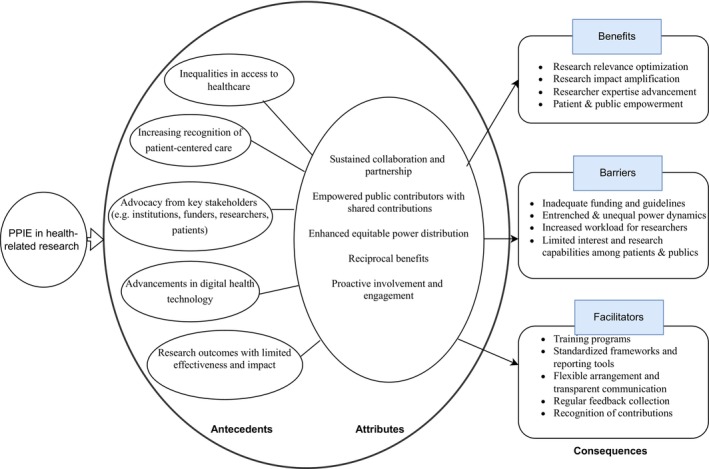
A Conceptual Model of PPIE Antecedents, Attributes, and Consequences (This figure describes the core components of the PPIE concept, including its antecedents, attributes, resulting benefits, and the identified barriers and facilitators related to its utilisation in health‐related research).

Figure 3 is a conceptual model outlining the key attributes, antecedents, and consequences of PPIE. In line with Rodgers' method, the concept analysis concludes with a model case, presented in Appendix S2. This hypothetical scenario synthesises the key findings of the study, including the definition, attributes, antecedents, consequences, and contextual elements of PPIE, and illustrates how the concept may be understood and applied in practice.

## 5 Discussion

This concept analysis applied Rodger's evolutionary framework (1989) to explore the definition, attributes, antecedents, and consequences of PPIE, drawing on 38 publications from diverse datasets. The notable findings are discussed below.

### 5.1 The Overall Evolution and Key Attributes of PPIE

Our findings indicate that the concept of PPIE is shifting toward greater clarity and consistency. Previous studies often used terms such as co‐design, co‐production, co‐creation, and patient and public involvement interchangeably, without clear distinctions or standardised definitions. The inconsistent use of these terms led to conceptual ambiguity and fragmentation, making it difficult to establish a coherent framework for meaningful public participation in research. PPIE has evolved beyond a narrow focus on patient inclusion, integrating these overlapping concepts into a structured and collaborative process (Jameson et al. 2023).

PPIE emphasises partnership, collaboration, empowerment, reciprocal respect and contributions, active involvement and engagement, as well as more balanced and equal power dynamics throughout the research process. Under the umbrella of PPIE, ‘involvement’ indicates a fundamental shift in how patients and the public are positioned in research (Spencer et al. 2023). Rather than occupying a peripheral role, patients and the public are embedded as integral partners across key research stages, where their knowledge, lived experiences, and insights are perceived to enhance the quality, relevance, and impact of research (Aiyegbusi, McMullan, et al. 2023). ‘Engagement’ under PPIE refers to activities such as disseminating research findings, raising awareness, organising public events, and increasing the impact of research. These activities are equally important, as they not only increase transparency and strengthen public trust but also broaden access to research and enhance its relevance for diverse communities (Hough et al. 2024). PPIE marks a broader transformation in research culture, positioning the contributions made by patients and the public as essential components rather than supplementary elements, and highlighting the transition of their role from passive consultation and tokenistic participation to active, sustained involvement (Hilton et al. 2024; Norrie et al. 2022).

### 5.2 The Primary Antecedents of PPIE

Our findings conclude that the emergence of the PPIE concept is driven by several antecedents associated with the needs of stakeholders (e.g., researchers, professionals, patients, and institutions), disparities in healthcare outcomes, and advancements in digital healthcare technologies. Researchers tend to enhance the applicability of their findings by incorporating diverse perspectives to address the limitations of traditional methods that often overlook patient and public input (Aiyegbusi, Cruz Rivera, et al. 2023; Croft et al. 2023). Healthcare professionals increasingly involve patients to deliver more personalised, patient‐centred care, recognising that professional expertise alone cannot meet the diverse needs of patients and highlighting the importance of shared decision‐making (Bensenor et al. 2022; Di Lorito et al. 2024; Hubbard et al. 2008). Patients are increasingly seeking a stronger voice in healthcare decisions, advocating for shared decision‐making and human‐centred care, and also expressing a desire to be involved in research processes (Fedorowicz et al. 2022; Gafari et al. 2024). International institutions, such as the NIHR, actively promote a paradigm that aligns research with public priorities, upholds participant rights, and maximises research impact (Aiyegbusi, McMullan, et al. 2023; de Graaff et al. 2021). The changing needs of stakeholders along with developments in healthcare delivery have contributed to a shift in how patients and the public are regarded, not only as recipients of care, but also as individuals whose experiences and perspectives are increasingly valued in other domains, including research.

On the one hand, health inequalities, such as unequal access to care, exclusion from research, and lack of influence in setting health priorities, have led to growing calls to involve patients and the public more directly in health research (Hanrahan et al. 2024; Hough et al. 2024; Karlsson and Janssens 2023). In this context, health inequalities have become one of the important antecedents driving the emergence and facilitation of PPIE to make research more inclusive and meaningful. On the other hand, involving people who experience health inequalities, particularly those from marginalised or underserved groups, can make research more relevant to their needs and experiences. This can help shift how priorities are set and how knowledge is produced, and may contribute to reducing health inequalities over time (Hawkes et al. 2023; Heaven et al. 2016). In addition, advances in digital health technologies, such as mobile health applications and online portals, enable real‐time interaction, reduce participation barriers, and allow patients and the public to engage in health research based on their own preferences, potentially addressing challenges like mobility limitations and geographic isolation (Weiler‐Wichtl et al. 2023). They also enable the collection and analysis of real‐time participant‐generated data, which can inform more personalised and responsive approaches to research execution and adaptation, enhancing the relevance of research across diverse contexts.

### 5.3 The Notable Consequences of PPIE and Its Utilisation

Utilisation of PPIE in health research faces continuing challenges. Resource constraints, such as limited manpower to carry out PPIE activities, time pressures, and insufficient funding support, as well as competing priorities among involved stakeholders (e.g., researchers, patients, public contributors), can undermine the sustainability of PPIE in health research (Heaven et al. 2016; Rolfe et al. 2018; Wyatt et al. 2024). Insufficient time allocated to the PPIE process and a lack of training opportunities constrain the ability of both researchers and public contributors to engage effectively (Simpson et al. 2018; Tolppa et al. 2024). Patients and the public face barriers such as low confidence in joining research endeavours, limited research skills, and uncertainty about their roles (Castillo et al. 2021; Preston et al. 2019). In addition, professional resistance, entrenched hierarchies, and the perception among some researchers that PPIE may fail to improve research quality can undermine and marginalise PPIE initiatives (Brett et al. 2014; Vargas et al. 2022). There is ongoing debate regarding the extent to which PPIE should be integrated across all phases of research or whether it has more applicability at specific stages. The absence of consensus on this issue poses challenges for researchers seeking to implement PPIE systematically and consistently. Furthermore, the lack of disease‐specific or context‐sensitive frameworks complicates efforts to adapt PPIE to the diverse needs and priorities of specific patient groups. For example, the design and application of PPIE strategies in chronic disease management may differ from those required in acute care settings or mental health services.

To effectively utilise PPIE in research, our findings highlight the importance of advanced preparation, including clear role definitions to establish expectations for researchers and public contributors (Gafari et al. 2024). Training in communication, research literacy, and cultural competence is essential to foster mutual understanding and collaboration (Karlsson and Janssens 2023; Zeissler et al. 2024). Equity in power dynamics should be prioritised by valuing public contributors' lived experiences and treating their insights as integral to the research process (Hawkes et al. 2023). That being said, challenges around power imbalances may persist, particularly regarding who holds decision‐making authority, whose perspectives are prioritised, and how contributions are recognised. In many cases, researchers are financially compensated for their participation; whereas public contributors may take part as unpaid volunteers. Although formal remuneration structures for public contributors do exist in some countries, such as the UK (NIHR 2021, 2022), these practices remain inconsistent across settings, raising ongoing concerns about fairness and long‐term sustainability. Future work should explore mechanisms to ensure more equitable involvement, such as shared governance structures, transparent decision‐making processes, and appropriate recognition or remuneration of public contributors to foster more meaningful and inclusive collaboration. For example, co‐governance structures can facilitate shared decision‐making on project goals, methods, and evaluation criteria (Fedorowicz et al. 2022). Transparency can be enhanced through regular feedback loops and sufficient resource allocation, including financial reimbursement and remote participation options to overcome barriers such as mobility and geographic constraints (Hough et al. 2024). Engaging underrepresented groups, such as ethnic minorities, individuals with disabilities, and those from lower socioeconomic backgrounds, is also critical. Strategies like partnering with community leaders, building trust with communities, and addressing cultural and linguistic barriers through plain language, translation services, or adaptive communication methods can improve accessibility and ensure diverse perspectives are represented (Aiyegbusi, Cruz Rivera, et al. 2023; Gafari et al. 2024).

## 6 Limitations

The application of Rodgers' evolutionary concept analysis framework in this study highlights the dynamic nature of concepts. However, this approach inherently allows for variability in interpretation due to its reliance on the selected data sources and the perspectives of the researchers. Additionally, it does not provide a definitive or operationalised model for the practical application of PPIE. Documents that placed greater emphasis on the implementation of PPIE, while giving less attention to its conceptual development, defining attributes, or antecedents, were excluded based on the aims of the study. As a result, the data collection may not fully encompass the scope of PPIE‐related studies or adequately represent its progression across varying contexts.

## 7 Implications

This study advances PPIE‐related theory development and deepens understanding of its core components, providing researchers and practitioners with clearer guidance on its definition and application in health research. The findings provide actionable insights into enhancing PPIE utilisation by identifying key barriers and facilitators while also supporting the establishment of standardised educational programs and training initiatives to promote effective PPIE practices. Healthcare and nursing researchers can use this study to clarify ambiguities in the PPIE concept and application, promote interdisciplinary collaboration, and better align research practices with the diverse needs of patients and the public. Over time, the continued integration of PPIE into nursing research may reinforce the profession's commitment to person‐centred care, ethical practice, and social accountability, while also contributing to the advancement of collaborative, inclusive, and contextually relevant research that informs and strengthens policy, education, and clinical practice.

## 8 Conclusion

PPIE has gained greater prominence in health‐related research for its contributions to research efficiency, relevance, impact, and innovation. However, inconsistent terminology and overlapping use of related concepts have caused confusion, limiting its proper application and alignment with established practices. A clear understanding of its definition, attributes, antecedents, and consequences is crucial for advancing its theoretical foundation, improving its implementation, and enhancing its impact on health‐related research. This study employed Rodgers' evolutionary concept analysis to clarify the concept and features of PPIE, examine its evolution over time, and identify facilitators and barriers to its utilisation. The study enables researchers and practitioners to gain a better understanding of what constitutes meaningful and effective PPIE.

## Ethics Statement

The authors have nothing to report.

## Consent

The authors have nothing to report.

## Conflicts of Interest

The authors declare no conflicts of interest.

## Supporting information


**Appendix S1:** jan70140‐sup‐0001‐AppendixS1.docx.

**Appendix S2:** jan70140‐sup‐0002‐AppendixS2.docx.

## Data Availability

Data will be provided upon request to the first author.
