# Supplementary material for: Evolution of Patient and Public Involvement and Engagement in Health‐Related Research: A Concept Analysis
Source: J Adv Nurs. 2025 Aug 16;82(5):4827–39. doi: 10.1111/jan.70140 (PMC13069254; doi:10.1111/jan.70140)
Supplement: Supplementary file 1 — Appendix S1: jan70140‐sup‐0001‐AppendixS1.docx. [file JAN-82-4827-s001.docx]

**Appendix 1: Definition, Antecedents, Attributes, Consequences, Contexts, and Related Terms of the PPIE Concept Identified from the Collected Documents**

| **No. Author(s)**  **Published year**  **Country**  **Context**  **Document type** | **Study Design** | **Evolution of PPIE covered in the document? (Y/N)** | **Involved stakeholders** | **How does the research conceptualize PPIE?**  **/** **Related terms mentioned in the research?** | **Attribute** | **Antecedent** | **Consequences** |
| --- | --- | --- | --- | --- | --- | --- | --- |
| Aiyegbusi et al. (2023)  UK  Context: Assessment of PPIE’s impact on therapies for long COVID in non-hospitalized individuals.  Review Article | Systematic Review | No | Individuals with long COVID, healthcare professionals; researchers and experts | Activities and research carried out with or by the public or patients, involving them in the development, execution, and management of projects, while gathering their insights and feedback on outcomes.  / Co-production | (1) Patients as co-applicants or co-authors.  (2) Patient collaboration with researchers in study design, dissemination of findings, and evaluation of the research. | 1. Growing recognition of the valuable contributions from patients and the public in research 2. Increasing support from international institutions to promote patient and public collaborations with researchers | **Benefits:** Research more relevant to the needs of patients and the public.  **Facilitators to the PPIE process:**   1. Diverse recruitment channels 2. Diversity and inclusion of patient partners 3. Structured PPIE groups 4. Adherence to standards and guidelines 5. Use of logs, discussions, and interviews for feedback analysis   **Barriers:**   1. Time and resource constraints in patient engagement 2. Misaligned patient insights and research goals 3. Patient stress 4. Management risks |
| Hanrahan et al. (2024)  UK  Context: The integration and impact of PPIE in the development of digital health technologies for neurological diseases*.*  Review Article | Rapid review | Yes | Patients and carers | Work that is done with or by patients and the public rather than to, about, or for them,  across the entire research cycle.  / N/A | (1) Patients and the public offer insights and contribute to research and practices.  (2) Patients and the public evaluate the real-world practicality of research and contextualize research more broadly. | (1) The need for patient-centered development is increasingly recognized.  (2) Thoughtful decisions by both researchers and users are essential for developing digital health technologies.  (3) International regulatory bodies prioritize patient involvement in qualifying digital health technologies | **Benefits:** Improve relevance, quality, and impact for the end users  **Facilitators to the PPIE process:**  (1) Active Patient Participation  (2) Diverse Engagement Methods  (3) Feedback Mechanisms  (4) Policy Support  (5) Listening to views on acceptability and feasibility  **Barriers:**  (1) Patient involvement mostly limited to one-off consultations, not continuous involvement  (2) Difficult to evaluate and report PPIE impact  (3) Not clear on the role of contributors in research |
| Clark et al. (2021)  UK  Context: PPIE evolution and application and inequities under Covid-19.  Commentary | N/A | Yes | N/A | Involving patients and the public in all research stages, from prioritization of topics to seeking research impact.  / N/A | (1) Researchers and contributors work across the project.  (2) Long standing collaboration. | 1. Inequalities in health 2. The COVID-19 pandemic offered an opportunity to gain a deeper understanding of the contexts of patient involvement. | **Benefits:** Improve equality and flexibility, support accessibility, and facilitate the relevance of research.  **Facilitators to the PPIE process:**  (1) Carers-carers’ engagement and third-sector community groups involvement  (2) Virtual meetings, community group’s newsletters and social media  (3) Participant awareness of research advantages  **Barriers:**  (1) Technology limitation  (2) Financial burden  (3) Complexity of academic settings |
| Aiyegbusi et al. (2023)  UK and USA  Context: PPIE in the drug development and regulatory processes.  Commentary | N/A | No | Patients/advocates, researchers, practitioners and drug developers | Activities are carried out with or by patients and the public, rather than to, about, or for them, promoting their active participation in regulatory science projects; research findings and outcomes are shared with patients and the public ensuring they remain informed.  / N/A | (1) Sustainable and structured interaction.  (2) Mutual learning for all parties.  (3) Effective collaboration. | (1) Strong commitment from regulatory bodies to implement structural and cultural changes.  (2) Ethical principles requirements.  (3) The COVID-19 pandemic led to increase public awareness of the importance of life sciences and the regulation of new medical interventions. | **Benefits:** Build trust in the regulatory process and improve the uptake of new medicines and technologies.  **Facilitators to the PPIE process:**  (1) Co-organize outreach events with patient partners in underserved areas.  (2) Ensure venues are accessible and well-connected.  (3) Reimburse contributors for expenses and time, and provide refreshments.  (4) Offer jargon-free training and flexible meeting options.  **Barriers:**  (1) Lack of consistency and systematicness during the implementation  (2) Lack of participants trust |
| Hough et al. (2024).  UK  Context: A PPIE group for individuals with hearing loss, aimed at sharing experiences, informing research, and improving patient services.  Research Article | Longitudinal study | Yes | Individuals with lived experience of hearing loss and/or cochlear implants | A collaborative approach involves conducting research with active public participation, fostering a two-way exchange of knowledge and mutual benefits.  / Patient and public involvement; Patient  led/user-controlled research; Co-production; Community engagement; Co-design | (1) Awareness that individuals have the right to influence research direction and methods.  (2) Consultation.  (3) Collaboration.  (4) Patient-led initiatives. | (1) Greater emphasis is being placed on public and patient involvement throughout the research process by academic communities.  (2) International funding bodies now require patient and public engagement in all funded studies. | **Benefits:** Boosts knowledge and fosters group belonging for participants; empowers members to create positive change; helps participant recruitment for researchers; generates new research ideas for researchers; contributes to research advancement; enhances skills in research and science communication.  **Facilitators to the PPIE process:**  (1) Ensures representation from various community groups.  (2) Structures for idea sharing and feedback between researchers and members.  (3) Relationship building and group administration.  (4) Ensure group diversity and inclusivity.  (5) Follows established guidelines.  **Barriers:**   1. Lack of diversity and inclusivity, resulting in an unrepresentative group in research. 2. Issues related to costs and expenses.   (3) Lack of awareness about the importance of PPIE. |
| Bensenor et al. (2022)  Brazil  Context: Analysis of PPIE in managing Atrial Fibrillation.  Commentary | N/A | Yes | Patients and community members | (1) Sometimes called Community Engagement and Involvement (CEI).  (2) Participants participate as co-applicants in all stages.  / N/A | (1) Active partnership involving people in a comprehensive way.  (2) Involving three pillars: public involvement, public engagement and participation. | (1) Global movements  (2) Social inequalities | **Benefits:**  (1) Understand comprehensive aspects, open new horizons and plan better health policies to meet public needs.  (2) Provide an opportunity to give patient and public a voice in setting research priorities and lead to better acceptance of research.  **Facilitators to the PPIE process:**  (1) Researchers be open-minded and listen to public voice.  (2) Make patient and public feel relaxed, encouraged and their opinions are fully considered.  **Barriers:**  (1) Researchers lack understanding from patient perspective.  (2) Support scarcity.  (3) Participant’s concern. |
| El-Nayir et al. (2024)  UK  Context: Analysis of PPIE in ASCEND PLUS Trail, assessing the effects of oral semaglutide on the primary  prevention of cardiovascular events in T2DM patients.  Research Article | Randomized controlled trial | Yes. | Type 2 diabetes patients | Activities and research carried out “with” or “by” members of the public or patients, rather than “to”, “about”, or “for” them and the findings can be shared with them.  / N/A | 1. Good participants experience 2. Maintained engagement and adherence of participants 3. Successful recruitment 4. Being protected safety and wellbeing of the participants | Resources support | **Benefits:** Optimizing the trial design, obtaining regulatory and ethical approval, and conducting the trial, ensuring the research relevance and acceptability.  **Facilitators to the PPIE process:**   1. Single contact person as the coordinator. 2. Innovative and streamlined trial design combined with digital technology support. 3. Ethnic diversity 4. High flexibility and adaptability of research process 5. Adequate resources(budget) 6. Experienced and professional researchers   **Barriers:** Tight timeline during trial |
| Fedorowicz et al. (2022)  UK  Context: The impact and process of using Facebook group in implementing PPIE in CVD risk management.  Research Article | Longitudinal study | No | Health service users, patients and clinic staff | Research is carried out ‘with’ or ‘by’ members of the patients, potential patients,  carers and all relevant stakeholders  instead of ‘about’ or ‘for’ them.  / N/A | 1. Patients and members of the public as actors undertaking or contributing to research. 2. Different research approaches have different PPIE needs. 3. Popular engagement times are 19:00-20:00 | (1) Funders, policymakers, and research organizations increasingly expectation.  (2) Various frameworks, guidelines, values and standards are developed by authority.  (3) The need to understand the local context, plan for future issues, and support inclusive practices. | **Benefits:** Providing user-friendly information and improving research relevance and quality by involving more diverse participants.  **Facilitators to the PPIE process:**   1. Facebook closed group or other flexible approaches combined with traditional approach. 2. Clear communication about the purpose of the group. 3. Dedicated time from research team and stable single coordinator. 4. Tailored strategy and set boundaries. 5. Continuous feedback from participants and rapid adjustment. 6. Increased convenience of participation.   **Barriers:**   1. Group maintenance due to time and resources constraints 2. Participants may not familiar with digital approach 3. Unrepresentative membership and has no specific inclusion criteria of participants |
| de Graaff et al. (2021)  Netherlands  Context: Analysis of PPIE in healthcare decision-making.  Commentary | N/A | No | N/A | PPIE not only as coproduction  but also as work that every initiative should be tailor-made.  / Co-production | Entails careful and inclusive relationship development that respects all participants and values their contributions. | Domestic and international research support. | **Benefits:** Improve the quality of decision making and increase fairness, responsiveness and legitimacy.  **Facilitators to the PPIE process:**   1. Increase the diversity of participants and reach specific group 2. Calibration and realignment of methodologies of participation 3. Value emotional way of participation   **Barriers:**  (1) Limited attention from policymakers and researchers.  (2) Lack of enthusiasm among patients and public.  (3) Issues regarding the representativeness of participants.  (4) Lack of clarity about the results.  (5) Considerable gap between ideal and practice.  (6) Uncritical promotion of PPIE.  (7) Difficulty in valuing all types of contribution. |
| Lorito et al.(2024)  UK  Context: PPIE in supporting dementia patients and carers in communities field by designing a co-design website.  Methodological Study | Participatory action research | Yes | Patients with dementia and carers | Involves actively engaging and including people with lived experience of the topic being studied. Unlike traditional research, which is done 'to,' 'about,' or 'for' patients and the public, PPIE emphasizes conducting research 'with' or 'by' them.  Visualized on a continuum that view knowledge as co-constructed through shared contribution.  / Co-design; Co-research | 1. Collaboration and partnership: public lived experience combined with academic knowledge 2. Ensuring respect and empathy 3. Ensuring equitable power distribution and fair access 4. Avoid using titles to address power dynamics | Founders request | **Benefits:**   1. For Researchers: Address public needs and priorities, enhance research relevance and quality, provide a platform for under-represented groups, and promote personal development. 2. For Participants: Build trust within the community, gain knowledge and inspiration. 3. Remote way enables wider involvement and anonymity.   **Facilitators to the PPIE process:**   1. Networking with community leaders 2. Developing terms of reference and rules of engagement 3. Investing adequate financial, labor resources and time for accessibility. 4. Equitable involvement, supportive environment and inclusive communication. 5. Personal contact and network of a experienced and reputable research team to build trust and relationship, offer personalized support. 6. Face to face interactions 7. Forward planning and clear agendas. 8. Attitudes, values and behaviors of all collaborators 9. Appropriate sampling method and s coping review of the literature 10. Combine external incentive (financial retribution) with intrinsic motivational strategy.   **Barriers:**   1. Lack of diversity 2. Participants felt unqualified or afraid to share. 3. Lack of effective incentive 4. Cultural barrier (e.g., gatekeeping by community leaders) 5. Pragmatic/ language/ literacy barrier 6. Excessive requirements for involvement. 7. Lack of access to key contacts. 8. People may lack of basic tech savviness. 9. Balancing pragmatic considerations with ethical concerns. |
| Forbat et al.(2024)  UK  Context: PPIE in care homes about palliative care.  Research Article | Longitudinal study | Yes. | One has relative live in care home, one did body check in care home, one is potential care home future user; researchers and clinicians | As a “spatial practice” and a mediating space for interaction and exchange, bringing together diverse knowledge and experiences from both users and researchers.  Participants involved as co-applicants.  / Co-production | 1. Power was dispersed among team members, leading to a self-governance approach. 2. Discussion sometimes felt more subjugating than collaborative 3. Complexity and dynamism of interactions. 4. Authenticity of participation varied from tokenism to co-production. 5. Public shared personal experiences; academics provided methodological knowledge. | 1. Consumerism focused on service improvement 2. Activist movements   Funding support | **Benefits:** Enhance processes and potential for impact, helping improve the relevance, quality and direction of research.  **Facilitators to the PPIE process:**   1. Egalitarian power relations 2. Trust, mutual understanding, ongoing communication and financial compensation. 3. Resources support 4. Recognition of reciprocity and different areas of expertise 5. Infrastructure(methods), clear onus and specific guidelines. 6. Understanding power dynamics and fostering a supportive environment and self-governing team. 7. Reflections on previous studies 8. Narrowed power differences and equality in the research team 9. Researcher’s attitudes about participants opinion   **Barriers:**   1. Inadequate pragmatic and emotional support. 2. Tokenism and unconscious bias. 3. Divergent understandings of roles leading to tension. 4. Tight timeframe. 5. Participants’ concerns about others’ understanding. 6. Balancing inclusivity with academic standards. 7. Polarized identities in the research team.   Exclusionary results due to academic nature. |
| Croft et al. (2023)  UK  Context: Discussion of the importance of PPIE as a “co-coordination capacity” for enhancing “absorptive capacity”.  Book chapter | N/A | Yes | N/A | Framed as involvement in evaluating, designing and implementing public service by professionals and the public’s collaboration as partners  / Co-ordination | (1) Offers the opportunity for stakeholders to bring new knowledge into service, creating the potential to enhance patients’ outcomes.  (2) Mainly embed at the general partitional practice level.  (3) Influenced by different forms of managerial control.  (4) Improve and lead decision-making about service design and delivery, helping develop more sustainable healthcare systems. | 1. Political demands of authority 2. Service- receivers need   Policy support | **Benefits:** Enhancing cost effectiveness and patient experience. And ensure provided service are responsive to public indeed need.  **Facilitators to the PPIE process:**   1. Formalized structures, systems and mechanisms. 2. Set up new legal requirements. 3. Internal and external networks. 4. Staff time, development and training. 5. Effective leadership and strategy. 6. Adequate budget. 7. Involvement at all stages. 8. Accessible knowledge sharing. 9. Mutual understanding with clear, effective and regular communication. 10. Education for participants 11. Effective integration of opinions. 12. Socialization capabilities. 13. Supportive cultural environment for diversity.   **Barriers:**   1. Lack of consensus of terminology and overlap structures of involvement 2. Professional hierarchies and managerial control 3. Professionals’ ignorance of public input. 4. Inadaptability of marginalized knowledge in local services. 5. Tokenism. 6. Lack of diverse involvement. 7. Rigid structures. |
| Karlsson and Janssens (2023)  Denmark  Context: The importance of introducing and involving PPIE into medical education.  Commentary | N/A | Yes | A mother from target populations | The process of developing health services and research with users of those healthcare services.  /Co-produce | 1. Reflect ethical values, such as patient rights. 2. Researchers and patient partners share reflections when engaged in coproduced health research. | Funders and journals requirements. | **Benefits:**  (1) Improved teaching that foster compassion and skill development, setting a positive precedent for careers.  (2) Enhanced understanding of disease and raised awareness of the research gap to ensure the research relevance and broaden studied areas.  (3) Aid counterbalancing epistemic injustice and increasing the person-centeredness in healthcare.  (4) Strengthened relationships between the health system and the public, fostering societal development.  **Facilitators to the PPIE process:**   1. Start teaching PPIE earlier in college and practice during education. 2. Respect participants' time. 3. Prioritize early reflection. 4. Support for educators.   **Barriers:**   1. Lack of proper role description, clear strategy, local level guidance, expectation alignment and material preparation. 2. Insufficient motivation, skills, and experience among researchers. 3. Inadequate infrastructure and resources. 4. Financial issues. |
| Gray et al (2021)  Australia  Context: Report of PPIE in nursing randomized controlled clinical trials.  Research Article | Descriptive study | Yes | N/A | Research carried out  “with” or “by” members of the public rather than “to”  “about” or “for” them.  /Co-design | Consumers are active partners in all aspects of research from generating research question to co-authoring publications. | Funding bodies support | **Benefits:** Improve research relevance, quality and impact.  **Facilitators to the PPIE process:**   1. Policy and guideline support. 2. Express gratitude to participants.   **Barriers:**   1. Identification difficulty. 2. Covid-19 pandemic. 3. Participants’ concern about speaking freely. 4. Risk of tokenism. |
| Hilton et al. (2024)  UK  Context: PPIE in community-based sleep management tool for dementia and mild cognitive impairment patients (TIMES).  Commentary | N/A | Yes | People living with dementia and people with caring experience | Research being carried out “with” or “by” members of the public rather than “to”, “about” or “for” them.  /Co-design | 1. An active partnership between patients, family and nonfamily carers, members of the public and researchers and they are shaping its direction actively in the research instead of participation. 2. Inclusive opportunities 3. Working together 4. Support and learning 5. Communications 6. Impact 7. Governance 8. Continuous and reflexive process that should be use in the whole process. | Epistemic justice theories | **Benefits:** Help research focus on participants practicalities and foster participants rapport and peer support.  **Facilitators to the PPIE process:**   1. Promoted diversity and inclusivity. 2. Collaborated with various institutions. 3. Supportive environment with flexible and considerable arrangement. 4. Member-led, Cooperative and formal structure. 5. Face to face interactions by having the research team visit participants. 6. Early, wider range involvement and maintain ongoing communication. 7. Combined participation way (offline and online). 8. Simplified communication and tailored information   sharing and training.   1. Reimbursement for participation. 2. Adequate time and financial resources.   **Barriers:**   1. Potential unconscious bias. 2. Diverse languages challenge. 3. Participants lack confidence to share. 4. Technology issues. |
| Hawkes et al (2023)  UK  Context: Reflection of PPIE in a behavioral intervention for people at T2DM risks.  Commentary | N/A | Yes: | Patients living with diabetes, at risk of diabetes, or had a family history of diabetes | Research carried  out ‘with’ or ‘by’ members of the public who are  actively involved in the research projects.  /Co-produce | 1. Contributors provide both personal knowledge and lived experience which complement the perspectives of the research team. 2. Should be tailored to the research nature. 3. Long timeframe and complicated programme. | International funders demand | **Benefits:** Enhance research quality and relevance for patients and carers by identifying research priorities, designing studies, and disseminating results to the public.  **Facilitators to the PPIE process:**   1. Clarified contributors’ role and expectation.   (2) Adequate training, resources and flexible timelines  (3) Early and continuous involvement in all stages of research.  (4) Use of standardized reporting tools.  (5) Open sharing and learning, respect for others  (6) Establishment of a steering committee.  (7) Simplified and accessible communication.  (8) Enhanced inclusivity and diversity.  (9) Multi-media dissemination  (10) Stable coordinator.  (11) Advance planning with contingency options.  (12) Regular feedback.  (13) Hybrid participation approach.  **Barriers:**   1. Time management challenge. 2. Inefficient dissemination of information. 3. The complexity of project itself and understand it within limited time. |
| Jameson et al (2023)  UK  Context: The development of  CHecklist for Inclusive Community involvement guidance among racially marginalized group.  Research Article | Qualitative study (Participatory Action Research+ case study) | Yes | Researchers and people from communities | Activity that is done ‘with’  or ‘by’ patients or members of the public rather than ‘to’  ‘about’ or ‘for’ them.  /PCIE (Patient, Public and Community Involvement and Engagement); PPI; Co-produce | Three stages:   1. building relationships 2. reciprocal relationships   practicalities of designing and (3) running community-based involvement activities. | Social inequalities and COVID-19 pandemic exacerbate it. | **Benefits:** Ensure equity in health research.  **Facilitators to the PPIE process:**   1. Build mutual trust and maintain ongoing relationship. 2. More informal meetings such as eating food together. 3. Regular feedback and considerable arrangement. 4. Standard reporting and practice guidelines. 5. Develop visual resources to help understand. 6. Adequate time, funding, and reimbursement for participants. 7. Flexibility and clear communication. 8. Designated coordinator and bilingual researchers. 9. Understand individual needs and open agendas.   **Barriers:**   1. Structural racism presence-lack of diversity. 2. Language and translation challenges. 3. Unfamiliarity with technology. 4. Limited participants pool. 5. The conflict between sustainable relationship and funding. |
| Hyde et al. (2017)  UK  Context: The process and impact of involving patients in systematic review and narrative synthesis of shared decision making around prescribing analgesia for musculoskeletal pain in  primary care consultations.  Review Article | Systematic review | Yes  . | Primary care physicians and non‐pregnant adults (over 18 years old) with MSK pain | Doing research ‘with’ or ‘by’ the public, rather than  ‘to’, ‘about’ or ‘for’ the public’, and where information and knowledge about research is provided and disseminated’.  /Shared decision making | (1) Can impact on research questions, methods, dissemination of findings and engagement with local communities.  (2) Methods of implementing PPIE range from consultation to collaboration. | Policy support | **Benefits:** Prioritizing research topics, clarifying language in invitation letters, providing a wider perspective in data analysis and designing outcomes more relevant to patients, increasing credibility and early identification of challenges in implementing findings.  **Facilitators to the PPIE process:**   1. Offer reimbursement 2. Use an established PPIE network. 3. Early and continuous involvement in all stages of research. 4. Adequate admin support. 5. Appoint a lead researcher and coordinator. 6. Flexible and considerable arrangement. 7. Training and clear communication. 8. Recognition of participants contributions. 9. Cite national and expert guidelines. 10. Address individual needs with tailored initiatives.   **Barriers:**   1. Tight time pressure. 2. Funding and time resources constraints. 3. Concerns about group dynamics (power imbalance). 4. Research ethics committee involvement. 5. Lack of diversity, leading to bias. 6. Insufficient understanding of PPIE members' feelings. |
| Branitsky et al (2024)  UK  Context: Co-design a digital application (VoiceIn) to support young people’s PPIE in mental health field.  Research Article | Qualitative research (Participatory Design) | Yes | Local young people, mental health professionals, PPIE representatives | Research should  be carried out “by” or “with” those who the research is  intended to benefit, rather than “to” or “for” them.  /Co-design | 1. Values the primacy of subjective lived experience in knowledge construction 2. Emphasizes a way of producing science where experts by experience are active co-designers and co-researchers through the entire research process | 1. Democratic deficit and power imbalance. 2. International policy | **Benefits:** Enhances research impact and promotes the exploration of novel ideas, improves research relevance, and ensures the alignment of studies with participants' priority needs. Face-to-face meetings also foster sustained and meaningful discussions, facilitating deeper exploration of concepts.  **Facilitators to the PPIE process:**   1. Digital health technology development. 2. User-friendly and flexible initiatives. 3. Regular feedback mechanisms. 4. Incentives or reimbursements. 5. Effective communication, training and clear role expectations.   **Barriers:**   1. Work and school demands and more limited independence and flexibility. 2. Limited interest or ability (due to digital inequalities) to attend meetings. 3. Resources (time and financial) constraints. 4. Rapid developmental changes among youth. 5. Challenges in engaging diverse participants. 6. Complexities in calculating remuneration for online participation. |
| Gafari et al (2024)  UK  Context: Strategies for engaging ethic minority in public health research based on community.  Commentary | N/A | Yes | Health community members | Research being carried out with or by members of the public, rather than to, about or for them.  /Co-production | 1. Members of the public are not sources of data but are collaborators, adding value to the research project. 2. The process takes time, needs true inclusion and reciprocity, is a two-way reach. 3. Researchers should be prepared to be uncomfortable about truth. | Health inequalities and exacerbated during COVID-19 pandemic.  Funding bodies demand | **Benefits:** Improve research quality, outcomes and applicability, and reduce research waste. Increase participants and researchers’ confidence, wellbeing and willingness to further engagement, create a virtuous circle of engagement.  **Facilitators to the PPIE process:**   1. Diverse group to contribute to all research stages. 2. Early start to recruitment of PPIE partners 3. Relationship-focused engagement. 4. Co-production and consultation activities. 5. Open communication and iterative feedback. 6. Co-production of project closure activities 7. Diverse research team and tailored research initiatives. 8. Adequate time and resources. 9. Dissemination of research findings.   **Barriers:**   1. Cultures and literacy levels differences with complexity of academic documents. 2. Conflicting priorities between researchers and public partners with researchers concerns. 3. Participants lack confidence and previous negative experiences. 4. Researchers inadequate experience and knowledge. 5. Inconsistency between guidelines and practice. 6. Lack of good practice examples and guidance. |
| Heaven et al (2016)  UK  Context: The Community aging research 75+(CARE 75+) study of PPIE in the elderly frailty.  Research Article | Cohort multiple  Randomised Controlled Trials | Yes | Elderly people across the frailty spectrum | Involving the public and patients in all aspects of research from design to dissemination.  /Public engagement; Co-production | (1) Cannot be prescriptive  and flexibility is needed.  (2) Focus on lay members  (3) Researchers prefer tokenism rather than build something bespoke. | 1. Moral, political, economic and pragmatic reasons. 2. Funders demand 3. Government’s commitment | **Benefits:** Raise researcher’s awareness of conducting meaningful research and ensure research relevance.  **Facilitators to the PPIE process:**   1. Monitoring and scrutiny. 2. Improved diversity. 3. Retention of lay representatives. 4. Clear role definition, training and fully recording. 5. Long standing relationships 6. Researchers positive attitude and supportive environment. 7. Regular feedback and flexible arrangements. 8. Support for staff 9. Simplified communication, avoiding academic jargon. 10. Collaboration with local networks.   **Barriers:**   1. Low recruitment rates 2. Lack of standardized guidance and structure. 3. High dropout rates 4. Resource constraints (manpower, time and budget) |
| Zeissler et al., (2024)  UK  Context: Evaluation study of PPIE’s impact on Parkinson Disease individuals.  Research Article | Mix methods study (Participatory Action Research+ Longitudinal Evaluation Study) | No | Patients and carers | A collaborative and adaptive process that includes co-developing programs with contributors requires customization to meet participants' unique needs and benefits from continuous learning based on past experiences.  /Co-design, co-developed | 1. PPIE recognized as a crucial factor in bringing key benefits to the entire life cycle of research projects. 2. Assessing the contributions of PPIE is essential for understanding its role and maximizing its value. | (1) Trials often miss recruitment targets due to restrictive criteria and lengthy follow-up, highlighting the need for PPIE.  (2) Few studies assess the impact of public involvement in healthcare research.  (3) Growing need for studies that implement and evaluate best practices for PPIE. | **Benefits:**  (1) Inclusive, sensitive research design increases accessibility, and has a positive impact on research deliverables.  (2) Ensures robust documentation and reliable data collection to effectively evaluate the impact of PPIE.  (3) Involving diverse contributors fosters rich discussions and broadens perspectives on topics.  **Facilitators to PPIE process:**  Ensure involving different voices  **Barriers:**  (1) Sharing insights from effective involvement in co-designing complex projects is limited.  (2) Face challenges on achieving a representative PPIE group.  (3) Face difficulties in involvement and engagement both in discussions and the decision-making process. |
| Wyatt et al. (2024)  North America and UK  A rapid umbrella review examines the involvement of children and young children in health research, and the associated benefits, challenges, and facilitators of each engagement.  Review Article | Rapid umbrella review | No | Children and young people | Promote research conducted 'with' or 'by' the public, rather than 'to', 'about', or 'for' them; not only 'consulted', 'involved' but also 'collaborated', and 'empowered' individuals.  /N/A | Seeking feedback from children and young people, actively engaging them in research, sharing responsibilities, and empowering them to lead various aspects of the process. | (1) There is a growing need for consistent terminology and detailed reporting of PPIE in the literature.  (2) Establishing an evidence base for best practices in involving children and young people in healthcare, policy, and advocacy is essential. | **Benefits:**  (1) Increased involvement enhances the relevance, completeness, and quality of research findings.  (2) Meaningful engagement fosters collaboration and maximizes research impact.  (3) Involvement offers financial compensation and age-appropriate skill development for CYP.  **Facilitators to PPIE process:**  (1) Use photovoice to enhance communication and improve research relevance.  (2) Involve youth from the start and secure realistic funding and resources.  (3) Ensure researchers are skilled in engagement practices and consult experts as needed.  (4) Involve youth in all stages of research and decision-making, ensuring their voices are prioritized.  (5) Create comfortable settings for open communication and adapt to youth needs.  (6) Design fun, age-appropriate tasks that allow youth to lead.  (7) Implement targeted strategies to maintain participant diversity.  (8) Set clear roles while involving parents to support youth engagement.  **Barriers:**  (1) Limited time, funding, and expertise hinder effective planning and implementation of PPIE activities, impacting engagement quality.  (2) Difficulty in achieving genuine power-sharing leads to distrust and perceptions of tokenism.  (3) Financial, scheduling, and transportation issues restrict participation, while loss of interest affects ongoing involvement.  (4) Small, homogenous samples limit engagement, particularly among young individuals who are often excluded from data access and sharing. |
| Weiler-Wichtl et al., (2023)  Europe  Context: Using different PPIE methods to assess knowledge and attitudes toward PPIE among pediatric oncology stakeholders in Europe.  Research Article | Mixed-method design (Participatory Action Research+ Cross-Sectional Survey) | No | Health care professionals patients, family members | Research conducted "with" or "by" members of the public, rather than "to," "about," or "for" them.  /N/A | Considering the needs of all relevant stakeholders, ensuring effective communication among subgroups, fostering collaborative relationships among all parties, optimizing the use of knowledge and resources, allow participants producing knowledge and create new research ideas. | (1) Awareness of early engagement in research is increasing, but gaps and disparities remain.  (2) Low familiarity with PPIE terminology among participants highlights a need for better education.  (3) Many European countries lack binding PPIE regulations despite existing guidelines. | **Benefits:**  Structured PPIE approach provides insights that enhance training tools and share findings with the pediatric oncology community, capturing perspectives traditional methods may miss.  **Facilitators to PPIE process:**  (1) Emphasize PPIE's importance, provide clear consent, and educate stakeholders on involvement options while enhancing public knowledge of research practices.  (2) Establish compulsory standards for patient involvement at all research stages.  (3) Ensure PPIE addresses patients' interests and needs.  (4) Improve tools for categorizing and analyzing qualitative responses from PPIE workshops.  **Barriers:**  (1) Insufficient awareness of PPIE and poor communication among stakeholders.  (2) Few effective tools for researchers to incorporate PPIE into the research process.  (3) Ambiguity in PPIE definition enhance complication.  (4) Results may be skewed by overrepresentation of individuals familiar with PPIE, mainly from patient organizations.  (5) Gender imbalance |
| Tolppa et al., (2024)  Pakistan  Context: The establishment of a PPIE group to support clinical trials in Pakistan, particularly in a lower-middle-income country setting within limited research infrastructure.  Commentary | N/A | Yes | Patients, families, carers, and community leaders | Collaborating with public members as active partners rather than subjects fosters meaningful participation. Providing personalized support ensures clear expectations, while using formal tools to document needs enhances relationship-building. Together, these practices highlight the importance of collaboration and effective communication in successfully implementing PPIE in research.  /N/A | Fostering broad engagement, maintaining transparency, and actively involving the public are essential for creating effective partnerships in PPIE, ultimately leading to more relevant and impactful clinical trials. | Even in countries with established support for patient and public involvement in research, such as the UK, Canada, and the US, direct participation in trial design and conduct remains limited. | **Benefits:**  The presence of PPIE groups with a broad remit can enhance early involvement by ensuring resources and expertise are readily accessible in all trial aspects.  **Facilitators to PPIE process:**  (1) increasing the availability of evaluations  (2) more case studies of best practices  (3) published more accounts on the effectiveness of various tools and approaches  (4) Ensure diverse outreach, flexible criteria, and the assessment of key characteristics to effectively engage individuals in the PPIE process  **Barriers:**  (1) Limited practice in low- and middle-income countries.  (2) Insufficient knowledge on setting up PPIE groups in resource-limited contexts. |
| Spencer et al., (2023)  UK  Context: Through the INSCHOOL project, investigating how the involvement of young people and co-production partnership in research will influence the relevance and quality of research outcomes.  Research Article | Qualitative methods (Participatory Action Research) | Yes | Young people with experience of the healthcare system as PPIE consultants, young people in secondary schools, and those living with a chronic disease | "no research about me without me" approach when designing studies in collaboration with young people.  Young people involvement often characterized by "involving" and "consulting" them, but direct participation in the research process, where they are allowed to "lead" or actively "support" research and knowledge creation, is less frequently observed.  /Co-design, co-production | PPIE goes beyond ethical obligations by providing vital insights and holding researchers accountable to individuals' lived experiences. It empowers young people to shape research questions and leads them in data collection through creative activities. PPIE advisors also contribute significantly to data analysis and the dissemination of findings. | Inconsistent terminology and blurred boundaries among research methodologies highlight the need for authentic collaboration. Researchers are urged to adopt sound and practical approaches to better integrate young people's perspectives into the research process. | **Benefits:**  (1) Empowering young people in the research process allows for greater control and more meaningful results.  (2) Involvement fosters confidence, builds research skills, encourages peer sharing, strengthens rapport with researchers, and leads to valuable contributions that enhance research knowledge and future care.  **Facilitators to PPIE process:**  (1) Require time and funding.  (2) Do thoughtful planning and adopt creative methods to encourage a participant-driven approach.  (3) Enhance participant engagement by promoting freedom of expression.  **Barriers:** (1) Inadequate support and preparation for PPIE.  (2) Power imbalances hinder the generation of knowledge and restrict the insights obtained from young people.  (3) Limited direct involvement of young people.  (4) Challenges in sensitive data collection. |
| Small et al., (2021)  UK  Context: Investigate how the collaborative co-design of tools will enhance the collection and use of patient experience data in healthcare. Research Article | Mix-methods design (Qualitative Study+ Co-design) | Yes | Individuals receiving treatment for serious mental health conditions, carers, and researchers | Involving members of the public, including patients and caregivers, in the research process itself, rather than conducting research on, about, or for them.  / Co-design, Co-developed, Co-production, Co-delivered | Establishing a dedicated group of public contributors, some of whom may act as co-investigators, enables input on study design from the outset. Co-design focuses on close collaboration between PPIE contributors and the research team through shared power, respect for individual knowledge, and the cultivation of reciprocal relationships to sustain collaboration. | (1) National Policy Encouragement to embed PPIE in research in the England.  (2) Framework and guidelines: A research institute established frameworks and promoted co-design, fosters meaningful partnerships that empower patient and carers in shaping research. | **Benefits:**  (1) Enhanced Adoption and Innovation.  (2) Improved understanding of user needs.  **Facilitators to PPIE process:**  (1) Tailoring PPIE to fit the preferences and values of research partners, considering individual needs for ongoing participation.  (2) Clarifying roles to effectively manage expectations.  (3) Ensuring transparency and sharing perspectives to enhance focused discussions and strengthen partnerships.  (4) Fostering peer relationships among PPIE contributors to improve retention and inclusivity.  **Barriers:**  (1) unexpected passing of PPIE contributors (underscores the emotional complexities of building long-term relationships in research).  (2) absence of necessary support and guidance  (3) difficult to provide adequate support within the group members  (4) Lack of Support for Emotional Challenges in Partnerships. |
| Simpson, Cook, & Miles (2018)  UK  Context: The increasing expectation and requirement for PPIE in health research and decision-making processes, particularly in the UK.  Research Article | Qualitative design (case study) | Yes | Patient and carers, clinicians | Using the frameworks established by INVOLVE, indicating a commitment to embedding PPIE into health research processes.  /N/A | 1. Identifying areas where mutually beneficial relationships can be built with patients and the public. 2. Adding value to outputs of HSRIC. 3. Ensuring accessibility and effective dissemination of outputs. 4. Ensuring that PPIE is strategic, meaningful, and appropriate. | The government-funded program INVOLVE, which supports active public involvement in health and social care research. | **Benefits:**  (1) leverages patient expertise to improve the likelihood of successful adoption and influence research priorities.  (2) improved user understanding.  **Facilitators to PPIE process:**  (1) Keep discussions on track and prevent distractions  from irrelevant topics.  (2) Ensure participants grasp the purpose and function of the EAA system to provide meaningful input.  (3) Navigate diverse personalities with care to foster a collaborative environment.  (4) Attention must be given to quieter members to ensure all opinions are represented and valued.  **Barriers:**  (1) Lack of accessible information about health technologies at early development stages.  (2) Difficulties for technology selection.  (3) Involving patients in prioritization is complicated due to the scarcity of information on emerging technologies. |
| Rouncefield-Swales et al., (2021)  UK  Context: the necessity for more robust evidence and documentation regarding the involvement of children and young people in PPIE within health research, including the methods used and the outcomes of their participation.  Review Article | Scoping review | No | Children and young people | Involvement means conducting research "with" or "by" members of the public rather than "to," "about," or "for" them, while engagement refers to the provision and dissemination of information and knowledge about the research.  /co-design, co-production, co-researchers, co-producers | Ensure diverse involvement (young participants can involve in research such as joining study design, data collection, analysis, and dissemination) | A ten-year plan from the National Institute for Health Research highlights the importance of public involvement in research, marking a significant shift toward integrating PPIE as a core component of research development. | **Benefits:**  (1) Benefits to participants: gain skills and confidence.  (2) Benefits to research: Involvement ensures relevant insights, user-focused tools, and effective dissemination.  **Facilitators to PPIE process:**  (1) Young participants participated sustainably as co-researchers and co-producers in the research.  (2) Providing training for researchers and enhancing activity design for accessibility.  (3) Engaging children and young people from the start to integrate their perspectives into planning and design.  **Barriers:**  (1) Difficulties to ensure meaningful participation of children and young people rather than tokenism.  (2) Lack of uniformity and transparency in reporting PPIE activities, leading to under-reporting and inconsistent quality.  (4) Challenges in attracting and keeping participants, as well as ensuring representative samples.  (5) Financial and resource limitations  (6) Difficulties in managing group dynamics and adapting to the personal circumstances of young participants.  (7) Misestimation of children and young people abilities. |
| Rolfe, Ramsden, Banner, and Graham (2018)  Canada  Context: Investigates how qualitative health research methods can improve patient and public involvement in health research, ensuring meaningful engagement and representation of diverse patient perspectives.  Commentary | N/A | Yes | Patients | Entails collaborating with 'patients'—a term commonly used in Canada and the US to encompass individuals, caregivers, and members of the public—to advance research in health and healthcare services. In this framework, patients are regarded as partners in the research process rather than merely as subjects or participants.  /N/A | The principles and practices of qualitative health research, such as relativism and social equity, align closely with the values and spirit of patient engagement. | Many research organizations remain in the initial phases of patient engagement, often depending on experiential methods instead of evidence-based practices, which highlights the foundational context for the development of PPIE. | **Benefits:**  Engaged patients and patient partners enhance the research process by contributing their insights at every stage, from formulating questions to disseminating findings.  **Facilitators to PPIE process:**  (1) Use diverse engagement methods like consensus voting and Delphi techniques.  (2) Apply member checking and constant comparative analysis for accurate findings.  (3) Provide summaries of patient recommendations.  (4) Adapt practices for ongoing partnerships with patient partners.  (5) Support transportation, childcare, remuneration, and training.  (6) Engage a diverse range of patients.  **Barriers:**  (1) Diverse patient experiences hinder quick decision-making in study design.  (2) Often tokenistic, minimally impacting research.  (3) Engaged patients may not reflect the broader population, affecting relevance.  (4) Many studies lack details about engagement processes, limiting effectiveness understanding. |
| Preston et al., (2019)  UK  Context: Explores PPIE in diabetes research at Queen Mary University of London and Barts Health NHS Trust, emphasizing partnerships with patients and the public to improve research and health outcomes, while addressing challenges and the need for ongoing evaluation and adaptation of engagement strategies.  Commentary | N/A | No | Diabetes patients, researchers, nurses, research coordinators and clinical physician | INVOLVE, the National Advisory Group formed by the National Institute for Health Research (NIHR) in 1996, characterizes PPIE as research conducted "with" or "by" public members, rather than research done "to," "about," or "for" them.  /N/A | Using a patient-centered approach; emphasize active partnership, offer constructive criticism, appreciate, and ensure a consistent meeting format for PPIE activities. | The establishment of the Lay Panel in 2007 as part of the Northeast London Diabetes Local Research Network have provided a structured platform for patient involvement in research. | **Benefits:**  Enhances research relevance by aligning studies with patient needs, fostering collaboration, raising awareness, and improving participant satisfaction, while also enriching knowledge and strengthening community ties.  **Facilitators to PPIE process:**  **(**1) Have effective PPIE strategy to enhance researchers' understanding of their investigations and build respectful community relationships.  (2) Have a careful planning on budgeting and resources  (3) Ensure clear communication to provide a brief study synopsis and up to five specific questions to the lay panel, clarifying requests and reducing workload.  (4) ensures all member responses are recorded and valued.  (5) Consider and listen to personal experiences.  (6) Ongoing planning to adapt to the evolving needs of research, enhance inclusivity for diverse populations.  **Barriers:**  (1) Lack of resources, necessitating budget allocations for administration, travel, hospitality, and meeting space.  (2) Inadequate preparation and training, resulting in overwhelming workloads for patients.  (3) Insufficient planning for the future. |
| Polanco et al., (2022)  European countries  Context: Discusses the concept of PPIE specifically in the field of pediatric oncology research  Commentary | N/A | No | Parents of children that had a history of cancer, or who currently had cancer, survivors of childhood, adolescent, and young adult cancers, health care professionals, academics and charity or support organisations | Promotes conducting studies 'with' the affected population rather than 'for' them.  /N/A | 1. Encompass enthusiasm, planning, practice, resilience, organization (represented by post it notes), and patience.   Research that incorporates PPIE has introduced valuable insights from real-world experiences into health research. | The varying definitions and goals of PPIE among stakeholders and cultures, coupled with the limited recognition of its benefits by researchers and healthcare professionals, underscore the need for clearer understanding and broader acceptance as essential antecedents to effective PPIE implementation in health research. | **Benefits:**  PPIE improves research outcomes and promotes meaningful stakeholder engagement.  **Facilitators to PPIE process:**  (1) Share findings through trusted social media and charity networks for better accessibility.  (2) Customized PPIE training improves representatives' effectiveness and collaboration.  (3) Formalized PPIE networks provide supportive frameworks for activities.  (4) Maintain collaborative partnership.  (5) Emphasize international data linkage from cancer registries with robust recording methods.  (6) Create standardized definition and guides for PPIE.  (7) Ensure opportunities for developing inclusive PPIE  practices exist at the grassroots level.  (8) Have positive reception of a European PPIE strategy.  (9) Gather information on existing PPIE activities to inform future strategies.  **Barriers:**  (1) Geographical Disparities: Variations in survival rates and limited access to treatments across Europe complicate clinical trial establishment.  (2) Knowledge Gaps: Lack of awareness regarding PPIE, its relevance, and defined levels.  (3) Infrastructure Deficiencies: Absence of necessary infrastructure and resources.  (4) Lack of standardization: Inconsistent definitions and practices for PPIE across Europe.  (5) Limited Representation: Narrow perspectives from workshop participant data.  (6) Healthcare Challenges: Difficulties in addressing low survival rates.  (7) **Research Misalignment**: Disconnect between research priorities and patient-focused perspectives. |
| Norrie et al., (2022)  UK  Context: A narrative review of literature and feedback from individuals with lived experience to identify effective practices and explore PPI infrastructures in health and care services for their applicability to the gambling support sector.  Review Article | Narrative review | No | Individuals with lived experience of gambling-related harm and gambling support services | PPI or PPIE are terms often used to describe the active and meaningful involvement of patients and the public in the planning, commissioning, delivery and evaluation of health services.  /co-produced/ co-productive/ co-production. | Provide choices and autonomy regarding the level and type of participation, promote inclusivity and accessibility, facilitate contributions to decision-making, offer regular feedback and recognition, and respect participants' time and expertise through appropriate remuneration. | PPIE is gaining recognition in public health and addiction services, but its use in gambling support is still limited. The Gambling Commission emphasizes involvement, and funding for the PWLE strategy reflects a commitment to PPIE principles, with emerging initiatives involving treatment participants in local services. | **Benefits:**  Establishes infrastructure that organizes the involvement of individuals with lived experience, identifying effective activities while respecting participants' desires, which broadens participation and ensures sustained engagement.  **Facilitators to PPIE process:** (1) Establish a National Forum to enhance involvement in gambling support and amplify lived experience voices.  (2) Recognize individuals with lived experience as key stakeholders.  (3) Create independent bodies to improve representation and facilitate decision-making involvement.  (4) Ensure recruitment into decision-making roles to guide research, education, and treatment.  (5) Secure commitment from organizations, along with adequate funding, support, and training for participants. **Barriers:**  (1) Limited exploration of effective practices for involving people with lived experience in gambling support services.  (2) Difficulty in recruiting a diverse range of participants  (3) Few documented cases  (4) Stigma around problem gambling hinders recruitment and participation. |
| Moult et al., (2023)  UK  Context: The evaluation of Patient and Public Involvement and Engagement (PPIE) activities within health and social care research funded by the National Institute for Health and Care Research (NIHR)  Research Article | Framework documentary analysis. | No | N/A | Characterized as research conducted "with" or "by" patients and community members, rather than simply "to," "about," or "for" them.  /co-produce, co-developed, co-designing. | (1) ‘Inclusive opportunities’, ‘Working together’, ‘Support and learning’, ‘Communications’, ‘Impact’, and ‘Governance’.  (2) fostering supportive environments, actively seeking feedback, learning from experiences, and displaying leadership in PPIE practices. | (1) NIHR requirement: The requirement for award-holders to report on PPIE activities and adhere to UK Standards establishes a structured framework that encourages public involvement.  (2) Engagement by Research Centers: The active engagement of research centers and their award recipients with local communities highlights their role in fostering innovative methods for involving patients and the public, which further underpins the practice of PPIE. | **Benefits:**  articulated in relation to the advantages experienced by public contributors, as well as the benefits observed at the organizational and project levels.  **Facilitators to PPIE process:**  (1) Integrate insights from public contributors effectively.  (2) Use the UK Standards and Insights framework to assess PPIE quality.  (3) Create platforms for collaboration and resource sharing among research centers.  (4) Ensure adequate outreach staff to enhance community involvement.  (5) Implement hybrid working models that combine online and in-person PPIE activities.  (6) Introduce independent assessments to minimize reporting bias.  (7) Collaborate with local and national partners to engage underserved communities and increase diversity.  (8) Focus on documenting both the successes and challenges of PPIE activities.  (9) Co-produce resources for secure management of public contributor information.  (10) Advocate for the NIHR Race Equality Framework to engage underserved communities.  **Barriers:**  (1) Difficulties in transitioning from in-person to online PPIE activities.  (2) Mergers and shifts in commissioning arrangements disrupt public involvement efforts.  (3) Challenges in ensuring effective PPIE within commercial research.  (4) Insufficient resources for sharing learnings and best practices.  (5) Difficulties in effectively reaching and engaging under-served populations.  (6) Have time constraints on building sustainable relationships.  (7) NHS restructuring and resource loss hinder public involvement initiatives. |
| Micklewright et al. (2024)  UK  Context: Engaging care home residents in PPIE through Activity Providers amplifies the voices of older adults, including those with cognitive impairments, and fosters meaningful changes in care practices while enhancing research quality.  Research Article | Exploratory design | Yes | Care home staffs, care home residents (dementia patients and older people) | Characterized by paying attention to the perspectives of individuals living in, visiting, or working in care homes, as well as the general public and other important stakeholders in the health and social care system, to improve the quality of research and increase the relevance of study findings.  / co-designing | Operating in an inclusive and participatory manner that incorporates diverse perspectives, sustaining partnerships, prioritizing the voices of residents, and ensuring meaningful engagement. | Care home residents are frequently left out as contributors to research that pertains to them, even in studies where PPIE is otherwise effectively considered and incorporated. | **Benefits:**  (Benefits to activity providers) reported gaining new skills and insights into residents’ preferences, allowing them to engage with residents at convenient times and in ways that meet individual needs. This collaboration enriches their professional development and enhances the overall quality of care.  **Facilitators to PPIE process:**  (1) Provide additional training for activity providers.  (2) Allocate more resources to evaluate and refine PPIE approaches.  (3) Effectively involve skilled groups to enhance the research process.  (4) Future studies are needed to explore the full potential of PPIE approaches.  **Barriers:**  (1) Time constraints make it difficult for facilitators to integrate PPIE into their routine work.  (2) Managing emotional feedback from residents complicates the engagement process.  (3) Recruiting and supporting activity pack facilitators requires significant resources, leading to continuity issues with personnel changes.  (4) Limited resident participation in topic selection and preparation can compromise the relevance of activities.  (5) Developing resources for unfamiliar residents is challenging and heavily relies on facilitator input.  (6) Direct collaboration with residents on activity packs is difficult, even with expressed interest.  (7) Gathering feedback through activity packs limits the ability to obtain direct insights from residents.  (8) Further research is needed to differentiate residents' thoughts from interpretations. |
| Loria-Rebolledo et al., (2023)  UK  Context: Examines how Covid-19's remote working shift impacts public participation in health research, seeking to identify preferences that enhance engagement and optimize PPIE effectiveness.  Research Article | Discrete choice experiment | Yes | Public contributors who have participated in research projects | Defined broadly as conducting research "with" or "by" public contributors, rather than "to," "about," or "for" them. This concept emphasizes active participation, and effective PPIE focuses more on co-production than mere involvement.  /co-producing | (1) Involve a range of activities to connect researchers and the public.  (2) Four pre-meeting attributes include meeting length, time of day, connectivity support, and technical assistance for participants.  (3) During meetings, two attributes focus on etiquette and the moderator's role.  (4) Lastly, one attribute addresses the provision of feedback on contributions after the meeting. | The lack of prior research on remote working quality in PPIE before the Covid-19 pandemic highlights a gap that the pandemic addressed with new guidelines. This context reveals how remote PPIE practices evolved, particularly regarding existing health inequalities and the pandemic's disproportionate impact on disadvantaged communities, emphasizing the need for diversity and inclusion. | **Benefits:**  Enhance participation for some contributors, making it more accessible. Additionally, flexible meeting formats, like remote options, promote project uptake and engagement.  **Facilitators to PPIE process:**  (1) Encourage feedback and flexibility to boost participation.  (2) Utilize a skilled moderator to ensure inclusive contributions.  (3) Involve public contributors in resource allocation decisions.  (4) Identify features that enhance participation, such as shorter meetings.  (5) Provide flexibility to improve contributor satisfaction.  (6) Limit the length of remote meetings to reduce participant burden.  **Barriers:**  (1) Varying participation levels depend on meeting features.  (2) Overlooked essential feedback for public contributors.  (3) Not all contributors have equal access to or comfort with virtual meetings.  (4) Contributors may feel their input is undervalued, decreasing motivation.  (5) Remote meetings limit nonverbal communication, impacting engagement. |
| Keane et al., (2023)  UK  Context: analyzes the demographics of participants in PPIE activities in the UK, emphasizing the need for greater diversity to enhance health research relevance and impact. Research Article | Descriptive study | Yes | Public contributors who have participated in vocal research projects patients, researcher, clinicians, anyone who will be influenced by the project | An active collaborative partnership between researchers and members of the public, patients, carers and/or communities, working alongside research  teams and as part of research organizations.  /co-production, co-applicants, co-creating, co-chaired, co-developed, co-led. | prioritizes research to meet the needs of those who will benefit most. It incorporates lived experiences, fosters collaboration, and provides training for researchers and contributors. It also raises public awareness, empowers participation, and integrates patient and public voices into research governance, supporting an engaged research culture. | (1) An urgent need for diverse and inclusive participation in PPIE, especially in the wake of the Covid-19 pandemic.  (2) Enhancing diversity is crucial for making health research relevant and beneficial to all segments of society.  (3) The lack of routine systems to collect and analyze demographic data highlights a significant gap that must be addressed to improve inclusion in PPIE. | **Benefits:**  Enhances the relevance, quality, ethics, and impact of research, contributing to high-quality outcomes. It fosters new skills and shifts in attitudes, while also improving recruitment and retention of participants in clinical trials. Additionally, collecting demographic data provides valuable insights for ongoing PPIE efforts.  **Facilitators to PPIE process:**  (1) Collect and analyze demographic data for inclusivity.  (2) Recognize qualitative and quantitative aspects of PPIE.  (3) Focus on specific communities to include diverse voices.  (4) Minimize overlap in engagement efforts.  (5) Control participation requests to avoid fatigue.  (6) Establish mechanisms for ongoing feedback.  (7) Keep up-to-date records of contributors and activities.  (8) Ensure staff involvement in the PPIE process.  **Barriers:**  (1) Lack of systems for routine collection and analysis of participant demographics in UK health research.  (2) Absence of standardized methods for capturing demographic and contextual information hinders inclusion.  (3) Participation challenges arise from low response rates, digital inequalities, survey duplication, and lengthy questionnaires, limiting the collection of detailed data. |
| Karlsson et al. (2024)  Denmark  Context: The integration of PPIE at a Danish university hospital reveals complexities in research culture, highlighting the need for changes in performance indicators and training to promote collaboration and inclusion. Research Article | Institutional ethnography | Yes | Patients, relatives, and researchers/clinicians | 'Research for and with patients and relatives'  /Co-creating, co-developed. | Engaging in active, meaningful, and collaborative interactions between patients and researchers throughout all stages of the research process, where decision-making is informed by patients' contributions as partners, acknowledging their unique experiences, values, and expertise. | Historical struggles of patients to gain well defined positions within the healthcare system.  build up knowledge on early context of a less context-sensitive approach, recognition on more understanding the local context and within this context to build knowledge of how organizations working with health research. | **Benefits:**  Highlights the need for changes in key performance indicators and training to improve collaborative research practices, making research more relevant to patients and easier to implement.  **Facilitators to PPIE process:**  (1) Understanding funders' expectations is crucial for initiating PPIE.  (2) More recognition the importance of patient involvement.  (3) Fostering institutional cultures that embrace PPIE is necessary for successful implementation.  **Barriers:**  (1) Lack of Embedding: PPIE is not yet integrated into standard research practices.  (2) Cultural Misalignment: Researchers often view PPIE as outside their roles and responsibilities.  (3) Conflicting Institutional Goals: Differing institutional priorities can obstruct effective PPIE implementation. |

**Reference list:**

Aiyegbusi, O. L., Cruz Rivera, S., Oliver, K., Manna, E., Collis, P., King-Kallimanis, B. L., , Bhatnagar V., Herold R., Hopkins J., Campbell L., Croker A., Leach M., Calvert M. J.et al. (2023). The Oopportunity for Ggreater Ppatient and Ppublic Iinvolvement and Eengagement in Ddrug Ddevelopment and Rregulation. Nature Reviews Drug Discovery, 22(5), 337–338. 10.1038/d41573-023-00031-x

Aiyegbusi, O. L., McMullan, C., Hughes, S. E., Turner, G. M., Subramanian, A., Hotham, R., , Davies E. H., Frost C., Alder Y., Agyen L., Buckland L., Camaradou J., Chong A., Jeyes F., Kumar S., Matthews K. L., Moore P., Ormerod J., Price G., Saint-Cricq M., Stanton D., Walker A., Haroon S., Denniston A. K., Calvert M. J., TLC Study Group, Brown K., Chandan J. S., Gkoutos G. V., Jackson L. J., Lord J. M., Marshall T., Marwaha S., Myles P., Nirantharakumar K., Rivera S. C., Sapey E., Simms-Williams N., Williams T., Wraith D. C.et al. (2023). Considerations for Ppatient and Ppublic Iinvolvement and Eengagement in Hhealth Rresearch. Nature Medicine, 29(8), 1922–1929. 10.1038/s41591-023-02445-x

Bensenor I. M. Goulart A. C. Thomas G. N. Lip G. Y. H. NIHR Global Health Research Group on Atrial Fibrillation Management 2022. Patient and Public Involvement and Engagement (PPIE): First Steps in the Process of the Engagement in Research Projects in Brazil. Brazilian Journal of Medical and Biological Research 55 e12369 10.1590/1414-431X2022e12369

Branitsky, A., Bee, P., Bucci, S., Lovell, K., Foster, S., & Whelan, P. (2024). CoDesigning a Digital App to Support Young People's Patient and Public Involvement and Engagement (VoiceIn): Development and Usability Study. JMIR Human Factors, 11, e53394. 10.2196/53394

Clark, M., van Vliet, E., & Collins, M. (2021). Reflections From the COVID-19 Pandemic on Inequalities and Patient and Public Involvement and Engagement (PPIE) in Social Care, Health and Public Health Research. Health Expectations, 24(5), 1547–1550. 10.1111/hex.13278

Croft, C., Currie, G., Kiefer, T., & Burgess, N. (2023). Patient and Public Involvement and Engagement (PPIE) for Enhancing Absorptive Capacity(ACAP) in Pursuit of High-Quality, Affordable and Equitable Healthcare. In Burgess, N. & Currie, G. (Eds.), Shaping High Quality, Affordable and Equitable Healthcare (93–111). Springer International Publishing AG. 10.1007/978-3-031-24212-0_5

de Graaff, B., Kleinhout-Vliek, T., & Van de Bovenkamp, H. (2021). In the Works: Patient and Public Involvement and Engagement in Healthcare DecisionMaking. Health Expectations, 24(6), 1903–1904. 10.1111/hex.13339

Di Lorito, C., Griffiths, S., Poole, M., Kaviraj, C., Robertson, M., Cutler, N., et al. (2024). Patient and Public Involvement and Engagement With Underserved Communities in Dementia Research: Reporting on a Partnership to Co-Design a Website for Post-Diagnostic Dementia Support. Health Expectations, 27(1), e13992. 10.1111/hex.13992

El-Nayir, M., Wijesurendra, R., Preiss, D., Mafham, M., Tsiotos, L., Islam, S., et al. (2024). Patient and Public Involvement and Engagement in the ASCEND PLUS Trial: Reflections From the Design of a Streamlined and Decentralised Clinical Trial. Trials, 25(1), 554. 10.1186/s13063-024-08393-2

Fedorowicz, S., Riley, V., Cowap, L., Ellis, N. J., Chambers, R., Grogan, S., , Crone D., Cottrell E., Clark-Carter D., Roberts L., Gidlow C.J.et al. (2022). Using Social Media for Patient and Public Involvement and Engagement in Health Research: The Process and Impact of a Closed Facebook Group.Health Expectations, 25(6), 2786–2795. 10.1111/hex.13515

Forbat, L., Macgregor, A., Brown, T., McCormack, B., Spilsbury, K., Rutherford, A., , Hanratty B., Hockley J., McKenzie M., Soulsby I., Ogden M.et al. (2024). Negotiating Pace, Focus and Identities: Patient/Public Involvement/Engagement in a Palliative Care Study. Sociology of Health & Illness, 46(7), 1327–1344. 10.1111/1467-9566.13785

Gafari, O., Bahrami-Hessari, M., Norton, J., Parmar, R., Hudson, M., Ndegwa, L., , Agyapong-Badu S., Asante K.P., Alwan N.A., McDonough S., Tully M.A., Calder P.C., Barker M., Stokes M.et al. (2024). Building Trust and Increasing Inclusion in Public Health Research: Co-Produced Strategies for Engaging UK Ethnic Minority Communities in Research. Public Health, 233, 90–99. 10.1016/j.puhe.2024.05.007

Gray R. Brasier C. M. Zirnsak T. M. Ng A. H. 2021 Reporting of Patient and Public Involvement and Engagement (PPIE) in Clinical Trials Published in Nursing Science Journals: A Descriptive Study Research Involvement and Engagement 7 1 88 10.1186/s40900-021-00331-9

Hanrahan, M., Wilson, C., Keogh, A., Barker, S., Rochester, L., Brittain, K., Lumsdon, J., & McArdle, R. (2024)/ How Can Patients Shape Digital Medicine? A Rapid Review of Patient and Public Involvement and Engagement in the Development of Digital Health Technologies for Neurological Conditions Expert Review of Pharmacoeconomics & Outcomes Research 25(2): 137-154 10.1080/14737167.2024.2410245

Hawkes, R. E., Sanders, C., Soiland-Reyes, C., Brunton, L., Howells, K., Cotterill, S., , Bennett C., Lowndes E., Mistry M., Wallworth H., Bower P.et al. (2023). Reflections of Patient and Public Involvement From a Commissioned Research Project Evaluating a Nationally Implemented NHS Programme Focused on Diabetes Prevention. Research Involvement and Engagement, 9(1), 42. 10.1186/s40900-023-00447-0

Heaven, A., Brown, L., Foster, M., & Clegg, A. (2016). Keeping It Credible in Cohort Multiple Randomised Controlled Trials: The Community Ageing Research 75+ (CARE 75+) Study Model of Patient and Public Iinvolvement and Engagement. Research Involvement and Engagement, 2, 30. 10.1186/s40900-016-0044-9

Hilton, A., Megson, M., Aryankhesal, A., Blake, J., Rook, G., Irvine, A., , Um J., Killett A., Maidment I., Loke Y., van Horik J., Fox C., TIMES programme teamet al. (2024). What Really Is Nontokenistic Fully Inclusive Patient and Public Involvement/Engagement in Research? Health Expectations, 27(2), e14012. 10.1111/hex.14012

Hough, K., Grasmeder, M., Parsons, H., Jones, W. B., Smith, S., Satchwell, C., , Hobday I., Taylor S., Newman T.et al. (2024). Patient and Public Involvement and Engagement (PPIE): How Valuable and How Hard? An Evaluation of ALL_EARS@UoS PPIE Group, 18 Months on. Research Involvement and Engagement, 10(1), 38. 10.1186/s40900-024-00567-1

Hyde C. Dunn K. M. Higginbottom A. Chew-Graham C. A. (2017) Process and Impact of Patient Involvement in a Systematic Review of Shared Decision Making in Primary Care Consultations.Health Expectations 20, 2:298-308 10.1111/hex.12458

Jameson, C., Haq, Z., Musse, S., Kosar, Z., Watson, G., & Wylde, V. (2023). Inclusive Approaches to Involvement of Community Groups in Health Research: The Co-Produced CHICO Guidance. Research Involvement and Engagement, 9(1), 76. 10.1186/s40900-023-00492-9

Karlsson, A. W., & Janssens, A. (2023). Patient and Public Involvement and Engagement (PPIE) in Healthcare Education and Thesis Work: The First Step Towards PPIE Knowledgeable Healthcare Professionals. BMJ Open, 13(1), e067588. 10.1136/bmjopen-2022-067588

Karlsson, A. W., Kragh-Sørensen, A., Børgesen, K., Behrens, K. E., Andersen, T., Maglekær, K. M., , Rothmann M. J., Ketelaar M., Petersen E. N., Janssens A.et al. (2024). Wider Institutional Research Cultures and Their Influence on Patient and Public Involvement and Engagement in Health Research: An Institutional Ethnography. Social Science & Medicine, 347, 116773. 10.1016/j.socscimed.2024.116773

Keane, A., Islam, S., Parsons, S., Verma, A., Farragher, T., Forde, D., , Holmes L., Cresswell K., Williams S., Arru P., Howlett E., Turner-Uaandja H., MacGregor I., Grey T., Arain Z., Scahill M., Starling B.et al. (2023). Understanding Who Is and Isn'’t Involved and Engaged in Health Research: Capturing and Analysing Demographic Data to Diversify Patient and Public Involvement and Engagement. Research Involvement and Engagement, 9(1), 30. 10.1186/s40900-023-00434-5

Loria-Rebolledo, L. E., Watson, V., Hassan, S., Gabbay, M., Tahir, N., Hossain, M., , Goodall M., Frith L.et al. (2023). Public Contributors' Preferences for the Organization of Remote Public Involvement Meetings in Health and Social Care: A Discrete Choice Experiment Study. Health Expectations, 26(1), 146–159. 10.1111/hex.13641

Micklewright, K., Killett, A., Akdur, G., Biswas, P., Blades, P., Irvine, L., , Jones L., Meyer J., Ravenscroft N., Woodhead H., Goodman C.et al. (2024). Activity Provider-Facilitated Patient and Public Involvement With Care Home Residents. Research Involvement and Engagement, 10(1), 7. 10.1186/s40900- 023-00537-z

Moult, A., Baker, D., Aries, A., Bailey, P., Blackburn, S., Kingstone, T., , Lwembe S., Paskins Z.et al. (2023). Using the UK Standards for Public Involvement to Evaluate the Public Involvement Sections of Annual Reports From NIHR Managed Research Centres. Research Involvement and Engagement, 9(1), 109. 10.1186/s40900-023-00517-3

Norrie, C., Bramley, S., Lipman, V., & Manthorpe, J. (2022). Transferable Learning About Patient and Public Involvement and Engagement in Gambling Support Services From Health and SocialCare: Findings From a Narrative Review and Workshop With People With Lived Experience. Journal of Integrated Care, 30(2), 189–202. 10.1108/JICA-06-2021-0030

Polanco, A., Al-Saadi, R., Tugnait, S., Scobie, N., Pritchard-Jones, K. (2022). Setting International Standards for Patient and Parent Involvement and Engagement in Childhood, Adolescent and Young Adult Cancer Research: A Report From a European Collaborative Workshop. Cancer Reports, 5(6), e1523. 10.1002/cnr2.1523

Preston, J. L., Berryman, V. R., Hancock, A., Pattrick, M., Worthington, A., Hitman, G. A., , Hood G. A.et al. (2019). Developing Patient and Public Involvement and Engagement (PPIE) in Diabetes Research: A Local Approach. Practical Diabetes, 36(3), 81–85. 10.1002/pdi.2220

Rolfe, D. E., Ramsden, V. R., Banner, D., & Graham, I. D. (2018). Using Qualitative Health Research Methods to Improve Patient and Public Involvement and Engagement in Research. Research Involvement and Engagement, 4, 49. 10.1186/s40900-018-0129-8

Rouncefield-Swales, A., Harris, J., Carter, B., Bray, L., Bewley, T., Martin, R. (2021). Children and Young People's Contributions to Public Involvement and Engagement Activities in Health-Related Research: A Scoping Review. PLoS One, 16(6), e0252774. 10.1371/journal.pone.0252774

Simpson, S., Cook, A., & Miles, K. (2018). Patient and Public Involvement inEarly Awareness and Alert Activities: An Example From the United Kingdom. International Journal of Technology Assessment in Health Care, 34(1), 10–17. 10.1017/S0266462317004421

Small, N., Ong, B. N., Lewis, A., Allen, D., Bagshaw, N., Nahar, P., , Sanders C., the DEPEND team, Hodgson D., Dehghan A., Sharp C., Dixon W., Lewis S., Kontopantelis E., Daker-White G., Bower P., Davies L., Kayesh H., Spencer R., McAvoy A., Boaden R., Lovell K., Ainsworth J., Nowakowska M., Shepherd A., Cahoon P., Hopkins R., Nenadic G.et al. (2021). Co-Designing New Tools for Collecting, Analysing and Presenting Patient Experience Data in NHS Services: Working in Partnership With Patients and Carers. Research Involvement and Engagement, 7(1), 85. 10.1186/s40900-021-00329-3

Spencer, B., Hugh-Jones, S., Cottrell, D., & Pini, S. (2023). The INSCHOOL Project: Showcasing Participatory Qualitative Methods Derived From Patient and Public Involvement and Engagement (PPIE) Work With Young People With Long-Term Health Conditions. Research Involvement and Engagement, 9(1), 91. 10.1186/s40900-023-00496-5

Tolppa, T., Hussaini, A., Ahmed, N., Dondorp, A. M., Farooq, S., Khan, M., , Masood A., Murthy S., Saleem S., Shuja Z., Zaman S., Hashmi M.et al. (2024). Establishment of a Patient and Public Involvement and Engagement Group to Support Clinical Trials in Pakistan: Initial Lessons Learned. Research Involvement and Engagement, 10(1), 98. 10.1186/s40900-024-00635-6

Weiler-Wichtl, L. J., Leiss, U., Gojo, J., Kienesberger, A., Hansl, R., Hopfgartner, M., , Schneider C.et al. (2023). Good to Know – This Is PPIE! Development of a Training Tool for Public and Patient Involvement and Engagement in Pediatric Oncological Research. Cancer Reports, 6(6), e1835. 10.1002/cnr2.1835

Wyatt, K. A., Bell, J., Cooper, J., Constable, L., Siero, W., Pozo Jeria, C., , Darling S., Smith R., Hughes E. K.et al. (2024). Involvement of Children and Young People in the Conduct of Health Research: A Rapid Umbrella Review. Health Expectations, 27(3), e14081. 10.1111/hex.14081

Zeissler, M. L., Bakshi, N., Bartlett, M., Batla, A., Byrom, D., Chapman, R., , Collins S., Cowd E., Deeson E., Ellis-Doyle R., Forbes J., Gonzalez-Robles C., Jewell A., Lane E. L., LaPelle N. R., Martin K., Matthews H., Miller L., Mills G., Morgan A., Parry M., Pushparatnam K., Ratcliffe N., Salathiel D., Scurfield P., Siu C., Whipps S., Wonnacott S., Foltynie T., Carroll C. B., McFarthing K., on behalf of the EJS ACT-PD consortiumet al. (2024). Patient and Public Involvement and Engagement in the Development of a Platform Clinical Trial for Parkinson's Disease: An Evaluation Protocol. Journal of Parkinson's Disease, 14(4), 809–821. 10.3233/JPD-230444
