# Supplementary material for: Evolution of Patient and Public Involvement and Engagement in Health‐Related Research: A Concept Analysis
Source: J Adv Nurs. 2025 Aug 16;82(5):4827–39. doi: 10.1111/jan.70140 (PMC13069254; doi:10.1111/jan.70140)
Supplement: Supplementary file 2 — Appendix S2: jan70140‐sup‐0002‐AppendixS2.docx. [file JAN-82-4827-s002.docx]

### ****Appendix 2: Model Case of PPIE and Its Utilization****

**Background**

Traditional research paradigms often fail to address the diverse needs of patients and the public, leading to inefficiencies, limited applicability of findings, and dissatisfaction among those they aim to serve. Increasing healthcare inequalities and the shift toward patient-centered care highlight the urgent need for inclusive and contextually relevant research approaches. Global advocacy for patient empowerment, alongside advancements in health technologies, has further encouraged active public involvement in research. In response, **Patient and Public Involvement and Engagement (PPIE)** has emerged as an innovative practice, integrating patients, caregivers, and community members across key research stages.

#### ****Case Overview****

A government-funded **breast cancer center** recently launched a **patient-centered care program** incorporating PPIE to ensure meaningful public involvement throughout the research process. The research team defined PPIE as a **collaborative process**, where research is conducted with and by patients and the public, extending beyond data collection to knowledge-sharing and community engagement. The team also introduced related terms, such as co-production, co-design, and co-creation, to clarify PPIE and highlight its evolution.

#### ****Recruitment and Participant Profiles****

The team recruited **seven public contributors** through hospitals, community outreach, and social media platforms:

1. **Ella-**60-year-old White retired woman, 10 years post-breast cancer surgery
2. **Emma**-45-year-old Black woman, housewife, undergoing chemotherapy for 5 years
3. **Linda**–35-year-old Asian working woman, recently diagnosed with breast cancer
4. **Andrey**–50-year-old male caregiver, supporting his wife for 2 years
5. **Anna**–25-year-old female caregiver, supporting her mother for 6 months
6. Maria-38-year-old female research nurse, work in public hospital cancer center for over 10 years and conduct cancer-related research for 5 years
7. Raven-31-year-old male staff (social worker) worked in local cancer patient organization (NGO) for over 3 years

#### ****Implementation of PPIE Principles****

To ensure **equity, inclusivity, and meaningful involvement and engagement**, the research team implemented the following strategies:

1. ***Defining Clear Roles and Responsibilities***
2. Developed a **PPIE handbook** for public contributors.
3. Provided **training sessions** to clarify contributors’ roles as **co-applicants, co-authors, and co-designers**, rather than passive participants.
4. For professional contributors, additional guidance was provided to help balance their expertise with patient-led priorities, so that their input complemented rather than dominated the discussions.
5. ***Ensuring Flexibility and Accessibility***
6. Adapted meeting schedules to accommodate contributors’ routines by offering **both in-person and online options**.
7. Compensated contributors for their time and reimbursed travel expenses.
8. ***Fostering Motivation and Collaboration***
9. Emphasized the **impact of PPIE**, such as: Improving breast cancer research relevance and effectiveness; influencing breast cancer care policies; enabling contributors to make informed healthcare decisions.
10. Reinforced **key messages** to promote collaboration: "We are working together toward the same goal: improving breast cancer care." "Your voice matters—there is no right or wrong answer. Just share your authentic thoughts." "This is a collaboration—your lived experiences lead the way."
11. Maria and Raven were encouraged to contribute their professional perspectives while keeping patient narratives at the core of the discussion, helping to foster balanced and inclusive dialogue.
12. ***Addressing Challenges Proactively***
13. Weekend focus groups were replaced with **flexible one-on-one sessions** due to chemotherapy-related fatigue.
14. Provided **psychological and practical assistance**, particularly for newly diagnosed patients and young caregivers.
15. Some participants hesitated to express opinions due to traditional beliefs that healthcare decisions should be made solely by professionals. To **dismantle hierarchical barriers**, the team organized **informal activities** such as shared meals and storytelling sessions.
16. To mitigate potential power dynamics between professional and non-professional contributors, ground rules were established (e.g., "Patients speak first," "No jargon").

#### ****Key Contributions and Impact****

Throughout the research process, public contributors played a **pivotal role** in shaping the study’s direction:

1. ***Research Design Adjustments***

Contributors identified a shared priority: **support for cancer survivors and caregivers: Ella** proposed a **psychological support module** for long-term survivors; **Emma** suggested **educational resources** for family caregivers; **Linda** advocated for **flexible care options** for working women; Maria contributed insights on clinical feasibility and research methodology, helping to align proposed interventions with the practical constraints of the healthcare system. Raven highlighted gaps in community-based support and helped connect the research to existing NGO resources for cancer patients.

1. ***Implementation and Evaluation***

The team **collaborated over several days** to develop an **integrated care plan** for cancer survivors and caregivers; the team conducted **interviews** to gather feedback on the proposed care plans; the team organized a **workshop** where public contributors presented findings to key stakeholders, raising awareness and providing actionable insights.

#### ****Outcomes and Long-term Impact****

PPIE fostered long-term trust between researchers and public contributors, laying a strong foundation for **future partnerships**. The proactive measures taken such as addressing **fatigue, emotional barriers, and power imbalances** ensured that all contributors felt **heard, valued, and empowered** throughout the process.
